# Selective spatial attention to competing speakers in azimuth and depth: Evidence from real and virtual environments

**DOI:** 10.1016/j.ynirp.2026.100390

**Published:** 2026-07-28

**Authors:** Benjamin Stodt, Daniel Neudek, Rainer Martin, Edmund Wascher, Stephan Getzmann

**Affiliations:** a Leibniz Research Centre for Working Environment and Human Factors at the TU Dortmund (IfADo), Ardeystraße 67, Dortmund, 44139, Germany; b Institute of Communication Acoustics, Ruhr-Universität Bochum, Universitätsstraße 150, Bochum, 44780, Germany

## Abstract

**Background and objective:**

Selective auditory attention enables listeners to focus on a target speaker while suppressing concurrent sounds. To assess the ecological validity of virtual reality for studying spatial attention, the present study investigated cognitive and neural mechanisms in both real and virtual laboratory environments.

**Method:**

Participants completed a selective spatial attention task in both real and virtual environments under auditory-only and audio-visual conditions while EEG was recorded. Speech stimuli were presented from different locations in both azimuth and depth. In each trial, two simultaneous speech stimuli originated from different locations. Each stimulus included a target or distractor word (plus visual patterns in the audio-visual condition) followed by a numeral. Participants indicated whether the numeral at the target location was odd or even, ignoring the competing numeral.

**Results:**

Performance was slower and less accurate in the virtual environment, with auditory-only stimulation, and in depth-only trials, suggesting slower accumulation of sensory evidence, as confirmed by drift-diffusion modeling. Early sensory ERPs (P1, N1) were largely stable across conditions, whereas P2 amplitudes were modulated by environment, stimulation, and spatial configuration. Spatial configuration also strongly influenced amplitudes of the contingent negative variation, which were largest with azimuth-only stimuli. Importantly, these effects were consistent across environments and largely independent of visual cues and significant cross-environment correlations were observed for all behavioral and neurophysiological measures.

**Conclusion:**

These findings suggest that, despite additional perceptual and cognitive demands indicated by reduced performance and slower evidence accumulation, virtual reality captures key mechanisms of selective attention.

## 1 Introduction

### 1.1 Spatial hearing and selective attention in multi-talker environments

Imagine trying to follow a colleague's voice in a busy open-plan office or a friend's story at a crowded social gathering. In such everyday situations, listeners must focus on one sound source while ignoring competing voices or background noise – a process known as selective spatial attention. It is mastered remarkably well by normally-hearing listeners and often referred to as the “cocktail-party effect” (Bronkhorst, 2015; Cherry, 1953; Haykin and Chen, 2005). Compared to localizing a single sound source in silence, these situations place greater demands on the auditory system (Darwin, 2008; Lewald and Getzmann, 2015). Furthermore, the challenge is particularly pronounced when the distractor is also speech, which leads to stronger masking effects and lower detection performance than with non-speech noise (Kidd et al., 2005). To manage such complex input, the auditory system appears to operate in distinct stages: first localizing and then selectively attending to the target speaker among competing sound sources. Initially, the system preattentively parses the mixture of sounds into distinct perceptual streams and meaningful auditory objects, primarily through bottom-up processes independent of the listener's attention (Bronkhorst, 2015). To achieve this, auditory input is initially analyzed in terms of relevant features (feature selection), such as intensity, frequency, pitch, or modulation characteristics. It is then organized into units based on physical and temporal similarity (feature grouping), whereby acoustically similar elements are combined into coherent auditory streams, whereas larger differences in acoustic features lead to their segregation into distinct perceptual sources (Bregman et al., 1990; Carlyon, 2004). Spatial hearing further enables the brain to exploit even subtle differences between the signals reaching the two ears, supporting processes that facilitate sound localization and source segregation (Blauert, 1996). Subsequently, higher-level (top-down) processes make use of context- and memory-based information, detecting patterns (such as predictable word sequences) to guide attention further (Kaya and Elhilali, 2017). This process, known as auditory scene analysis, constitutes a fundamental preattentive mechanism, allowing listeners to efficiently separate and organize auditory information before attention is directed (Bregman, 1990; Bregman et al., 1990; Sussman, 2005). After organizing the scene into separate auditory objects, attention may be divided across multiple streams if several sources are relevant or focused on a single target while suppressing irrelevant ones (Alain and Arnott, 2000; Sussman, 2017). Once established, auditory object segregation is largely maintained, while the system continuously monitors for changes or new streams that may require reanalysis (Sussman, 2017).

As introduced above, the discriminability of auditory objects increases with greater differences in their acoustic characteristics, which serve as important cues for determining their spatial positions. Research has extensively examined the mechanisms of selective spatial attention in scenarios where multiple speakers are arranged at different azimuths, separated by varying angular distances (e.g., Bidelman et al., 2025; Feng et al., 2025; Getzmann and Näätänen, 2015; Lewald et al., 2016; Lewald and Getzmann, 2015; for review, see Bronkhorst, 2015). Such setups are relatively straightforward to implement in the laboratory, either by arranging two or more speakers around the participant or by presenting auditory stimuli through headphones using binaural rendering. In contrast, fewer studies have investigated scenarios in which sound sources are distributed more widely in space, including variations in depth relative to the listener. This leaves open questions about how the auditory system processes spatial information and allocates attention in listening environments that include variations in both azimuth and depth relative to the listener (Kolarik et al., 2016; Mathiak et al., 2003; Paquier et al., 2016; Zahorik et al., 2005). Sound sources along the azimuth provide robust binaural cues, such as interaural time (ITD) and level differences (ILD), that support both the localization and segregation of sounds and enhance signal detection in noisy environments (Blauert, 1996). In contrast, differences in depth alone, relying on cues such as overall intensity, the direct-to-reverberant ratio, or spectral changes, are typically less salient, making it harder to distinguish concurrent speakers (Brungart and Simpson, 2002; Kolarik et al., 2016; Lavandier & Prud'homme, 2025; Zahorik, 2002; Zahorik et al., 2005). A reduced salience of depth cues can increase the cognitive and perceptual demands on the auditory system, especially in environments with multiple concurrent speakers, making it a critical factor in understanding selective spatial attention in real-world listening. Therefore, having at least one speaker along the azimuth is likely to facilitate selective attention, whereas discrimination becomes more challenging when both speakers vary primarily in depth relative to the listener. Additionally, listeners comprehend speech more accurately in multi-talker situations when the target speaker is spatially separated from competing sounds, an effect known as spatial release from masking (Hawley et al., 1999; Ihlefeld and Shinn-Cunningham, 2008; Noble and Perrett, 2002).

It has been shown that auditory localization accuracy can improve when the sound source is also visible to the listener (Shelton and Searle, 1980). Moreover, visual guidance, such as a brief cue presented shortly before or simultaneously with the auditory target at its spatial location, has been shown to enhance target discrimination and localization (Best et al., 2007; Getzmann and Wascher, 2017). These findings suggest that the brain can integrate auditory and visual information to optimize spatial attention, with visual cues potentially facilitating the segregation and selection of relevant auditory streams in complex environments.

### 1.2 Neural correlates of selective spatial attention

Successful speech perception in cocktail-party situations depends on both divided and focused attention (Shafiro and Gygi, 2007). A common way to study this interplay is to present multiple concurrent speakers, requiring participants to first detect a target word among the competing voices before attending to the corresponding speaker. During the divided attention phase, all speakers are monitored in parallel to select the relevant one, while in the focused attention phase the listener must concentrate on the target speaker suppressing irrelevant speech. Getzmann and colleagues (2011, 2014, 2016) introduced different versions of a “stock-price monitoring task”, in which listeners monitored a target company within sequences of company names and simulated stock prices. In this task, the company name served as a cue indicating the spatial location of the relevant information, while the stock price served as the target that determined the response. Specifically, focused attention was assessed by requiring participants to press a button whenever the stock price following the target company was equal to a specific value or within a predefined range. Neurophysiological activity was simultaneously recorded using electroencephalography (EEG). Getzmann et al. (2016) demonstrated that preparatory attention, indexed by an anticipatory slow wave known as the contingent negative variation (CNV) occurring between the cue and the target stimulus, enhances the accurate identification and monitoring of target speech in the presence of competing speakers. The CNV is one of the earliest investigated event-related potentials and was initially associated with processes of expectancy and response preparation (Walter et al., 1964). It is typically largest over fronto-central sites, consistent with involvement of medial frontal regions such as the cingulate and supplementary motor areas in anticipation and response preparation (Aydin et al., 2022; Gómez et al., 2007). Later research has demonstrated that it also reflects higher-order cognitive processes, including the preparation for an upcoming event and the orienting of attention toward task-relevant stimuli (Brunia et al., 2011; Brunia and van Boxtel, 2001; Kononowicz and Penney, 2016). While a reduced CNV amplitude is observed when selective attention to a task-relevant stimulus is impaired by competing stimuli (Tecce and Scheff, 1969), larger amplitudes are associated with more effective attention allocation and improved target detection, making the CNV a valuable marker of how anticipatory neural activity and attentional orienting support perception and behavior.

Earlier auditory ERPs than the CNV, such as those within the P1-N1-P2 complex, are typically characterized by maximal amplitudes over fronto-central scalp regions and reflect successive stages of auditory cortical processing following stimulus onset. More specifically, the P1 is primarily associated with the early sensory processing of the auditory input's physical characteristics and early orienting to attended sounds (Eggermont and Ponton, 2002; Grunwald et al., 2003), whereas the N1 reflects cognitive and attentional processes, signifying the initial stages of attentional focus and orientation toward task-relevant stimuli, with enhanced amplitudes for attended stimuli (Hillyard et al., 1973; Lange, 2013; Näätänen and Picton, 1987). Besides, the P2 is associated with higher-level processing such as stimulus identification, categorization, and relevance evaluation (Arnott et al., 2011; Potts, 2004). Overall, these potentials provide a useful index of early, preattentive auditory processing, allowing us to examine how spatial attention operates when listeners focus on a target voice within an auditory scene.

A previous study on auditory spatial change detection found no amplitude differences in the P1, N1, or P2 components when stimuli shifted either along the azimuth or depth dimension, suggesting that early neural processing operates similarly across both spatial dimensions (Stodt et al., 2025). However, as the auditory stimuli in that study were presented in isolation, it remains unclear whether similar neural patterns would emerge under more complex listening conditions. In these situations, increased demands on auditory segregation and selective attention might alter early sensory processing.

### 1.3 Virtual reality as a tool for studying selective spatial attention

Previous investigations of cocktail-party or multi-talker scenarios have typically relied on simplified laboratory setups. While such approaches ensure a high degree of experimental control, they offer limited ecological validity and may not accurately capture the complex spatial and sensory dynamics of real-world listening situations. Virtual reality (VR) enables the implementation of scenarios that are difficult or impossible to realize in conventional laboratories, while maintaining high levels of control and standardization (de la Rosa and Breidt, 2018; Keidser et al., 2020; Minderer et al., 2016; Parsons, 2015; Thurley, 2022). For instance, VR allows researchers to simulate complex auditory scenes such as a busy office, a café, or a classroom, where multiple speakers and environmental noises occur simultaneously, enabling controlled investigation of attentional mechanisms in settings that closely resemble everyday listening conditions (Breuer et al., 2022;Valzolgher et al., 2024). Some studies have shown that laboratory findings can be replicated in VR, indicating its potential for auditory research within more naturalistic scenarios. Previous studies have assessed spatial perception in auditory and audio-visual VR using a variety of behavioral paradigms and outcome measures rather than a standardized evaluation framework. Most commonly, spatial perception has been evaluated through sound localization tasks, measuring localization accuracy in terms of azimuth and elevation error, front-back confusions, or perceived externalization of virtual sound sources (e.g., Begault et al., 2001; Majdak et al., 2010; Parseihian et al., 2014; Piceda et al., 2025). Other studies have focused on distance perception and the ability to discriminate sound sources at different depths (e.g., Lin et al., 2024; Stodt et al., 2024). In addition, some investigations have employed cross-modal spatial alignment tasks (Roβkopf et al., 2025), assessed spatial perception through speech intelligibility measures (e.g., Guastamacchia et al., 2025; Ramírez et al., 2024), reaction times (e.g., Loushy Kay et al., 2025; Moraes et al., 2023), or training-related improvements in localization performance (e.g., Balan et al., 2017). Together, these studies suggest that virtual environments can provide sufficiently realistic spatial information for a range of experimental applications, while performance depends strongly on the rendering approach and the availability of spatial cues, with distance perception often remaining more challenging than azimuthal localization. However, a substantial proportion of these studies evaluated spatial perception exclusively within virtual environments, without direct comparison to real-world reference conditions, limiting conclusions regarding cross-environment correspondence.

Although VR represents a first step toward ecological validity, it still requires further refinement to fully approximate real-world conditions (Balboa et al., 2024; Hohmann et al., 2020; Kalantari et al., 2021, 2024; Pappalettera et al., 2024; Paquier et al., 2016; Rumiński, 2015; Stodt et al., 2024, 2025). As Thurley (2022) points out, researchers should consider that neural responses and behavior in virtual setups may differ from those in real environments. Moreover, not every experimental scenario is equally suited for VR implementation. Therefore, before VR can be fully established as a tool for investigating selective attention under more naturalistic conditions, it is essential to determine whether behavioral and neurophysiological findings obtained in virtual environments are comparable to those observed in comparable real-world settings. Demonstrating such comparability is critical for ensuring that results from VR studies can be reliably interpreted and generalized to real-world contexts.

### 1.4 Aims and hypotheses

The primary aim of the present study was to evaluate the validity of VR as a tool for investigating selective spatial attention in complex auditory scenes by examining the degree to which behavioral and neurophysiological measures obtained in a virtual environment correspond to those obtained in an equivalent real-world setting, and whether established effects of selective spatial attention can be replicated across environments. Demonstrating such correspondence across real and virtual environments would support the use of VR as a valid research tool for investigating selective spatial attention in more complex and ecologically valid spatial settings, whereas systematic differences could highlight potential limitations of virtual setups and provide insights into how virtual contexts may alter sensory processing and attentional mechanisms.

To address this aim, we compared a cocktail-party scenario in a reverberant real environment and a corresponding virtual environment, with two speakers presented at different spatial locations varying in azimuth or depth relative to the listener. Participants performed a selective spatial attention task in which they first localized a target word among two concurrent speakers and then attended to the target speaker's position to respond to the spoken numeral. We expected behavioral and neurophysiological measures obtained in the virtual environment to closely correspond to those obtained in the real environment, reflected in significant positive correlations between participants' performance and neural indices across environments.

In addition, we examined whether established effects of spatial configuration and visual stimulation on behavioral and neurophysiological measures would be replicated across both real and virtual environments. Specifically, we hypothesized that performance would be better when at least one speaker was presented along the azimuth dimension, compared with scenarios in which both speakers were positioned at varying distances in front of the listener, where binaural spatial cues are less salient. To further understand the cognitive mechanisms underlying target selection in multi-talker scenarios, we also applied drift-diffusion modeling to the behavioral data, allowing us to separate effects on decision speed, accuracy, and evidence accumulation.

We further examined neurophysiological correlates of selective attention. Here, we expected that the P1-N1-P2 complex, as a marker of early auditory processing, would be modulated by the spatial configuration of competing speakers, providing insights into how initial sensory encoding and attentional allocation are influenced by spatial attention demands. We further expected a CNV occurring between the onsets of the cue and the target stimuli, reflecting preparatory activity triggered by the cue that facilitates processing of the subsequent target through allocation of attentional resources. Since higher CNV amplitudes are associated with improved target detection, while reduced amplitudes reflect impaired selective attention due to interfering distractors, we expected the CNV to vary with the spatial configuration of the speakers. Specifically, the CNV was expected to be largest when at least one speaker is positioned along the azimuth, where robust binaural cues facilitate the segregation of auditory objects, and smallest when both speakers are located in depth, where these cues are weaker or absent, increasing the demands on selective attention.

Finally, we explored whether the availability of additional visual spatial cues modulates auditory selective attention. We expected that visual information co-located with the auditory target would facilitate attentional selection and improve behavioral performance, as well as modulate early and anticipatory neural responses.

## 2 Materials and methods

### 2.1 Participants

Data of 24 participants (14 female, 10 male), aged 18-32 years (*M* = 25.04, *SD* = 3.69), were included in the final analyses. During data collection, five additional participants were excluded based on the following predefined criteria. Two participants did not complete the second experimental session and were therefore excluded from all analyses. Three participants were excluded due to performance at or near chance level, indicating that they did not adequately understand or follow the task instructions. These exclusion criteria were applied uniformly and prior to data analysis. No further unreported experiments or negative findings relevant to the research questions were identified in this study.

The experiment was conducted across two testing sessions (one in the real and one in the virtual environment in randomized order) typically spaced 14 days apart. On each session, participants received standardized written instructions on the upcoming task. Inclusion and exclusion criteria were predefined prior to recruitment and applied consistently across all participants to reduce selection bias. All participants reported normal hearing and vision, no history of neurological disorders, and no use of medication that could have influenced the experiment. Prior to the first session, pure-tone audiometry was conducted (Oscilla USB 330; Inmedico, Lystrup, Denmark) using frequencies from 125 Hz to 4000 Hz to screen for hearing sensitivity and ensure no relevant hearing impairments were present. All participants were right-handed.

The study was conducted in accordance with the ethical standards of the Declaration of Helsinki and was approved by the Ethics Committee of the Leibniz Research Centre for Working Environment and Human Factors in Dortmund, Germany. Written informed consent was obtained from all participants prior to the experiment. Participants received monetary compensation or equivalent course credits.

### 2.2 Real environment

**Fig. 1:**
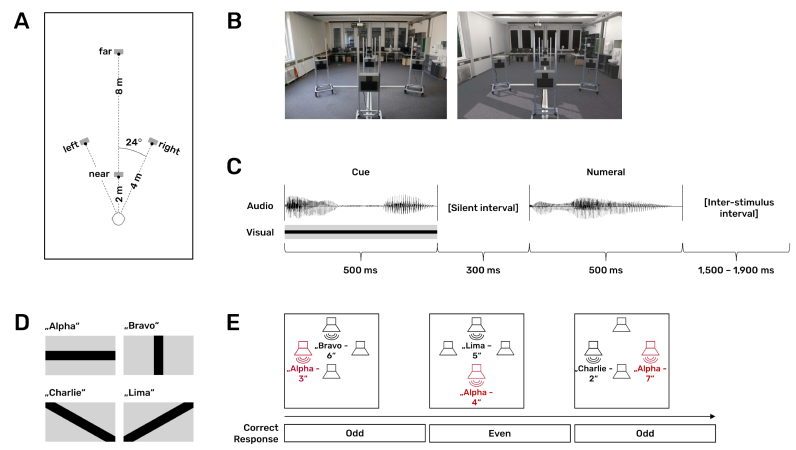
(A) Top-down view of the participant seated in front of four loudspeakers positioned at different locations in the room. (B) Real laboratory environment from the participant's perspective (left) and corresponding virtual environment presented via head-mounted display (right). (C) Temporal structure of a stimulus, consisting of a word and a numeral (here: "Alpha – 3″ of female speaker). (D) Visual stimuli displayed simultaneously with the cue word in the audio-visual condition. (E) Example of a trial sequence from the selective spatial attention task. Participants were instructed to determine whether the numeral presented at the target location (red marked) – indicated by the preceding cue (e.g., “Alpha”) – was odd or even, while ignoring a concurrent numeral.

The experimental room was the same as described in Stodt et al. (2024, 2025). It featured primarily concrete walls, a carpeted floor, and large windows on the left and rear sides, resulting in a mid-frequency reverberation time (RT60, averaged across 1/3-octave bands from 400 to 1250 Hz) of approximately 0.91 s and a critical distance of *d*_*c*_ ≈ 3.5 m. The room measured 7.22 m in width, 12 m in length, and 3.4 m in height. Participants were seated on a height-adjustable chair positioned centrally between the left and right walls and 2 m away from the rear wall. Four wooden boxes, each housing a loudspeaker (Visaton SC 8 N, 8 Ohm, 8 cm) and a computer screen (Samsung QB13R, 12.9″) mounted below it, were positioned in front of the participants: two boxes were placed along the median plane at distances of 2 m (near) and 8 m (far), while two additional boxes were positioned to the left and right at ±24° azimuth at a distance of 4 m from the listener (see Fig. 1A). Additionally, an identical box was placed at a central position 2 m behind the near and in the middle of the left and right boxes. This central box's loudspeaker and screen were inactive during the task but featured an orange fixation point for participants to focus on throughout the task. Each loudspeaker was mounted at a height of *h*_*LS*_ = 1.14 m, with the screens positioned directly below at a height of *h*_*S*_ = 0.99 m (center of the screen). Participants were seated such that their ear level was approximately *h*_*P*_ = 1.80 m. This configuration was required to ensure an unobstructed direct sound path from all loudspeaker positions to the listener, thereby maintaining consistent acoustic conditions across spatial positions. To ensure consistent head position and gaze direction throughout the experiment, a chinrest was used.

### 2.3 Virtual environment

While participants remained seated in the same position as in the real environment, a head-mounted display (HMD; HTC Vive Pro 2) was used for presenting the virtual environment combined with external open designed headphones (Beyerdynamic DT990) for audio playback. The visual representation of the laboratory room was created in Blender, accurately modeling its real dimensions along with all objects and furniture, using realistic textures. The scene was rendered, and experimental control was managed using Unity (Bebko and Troje, 2020). A direct visual comparison of the real and virtual environments is shown in Fig. 1B. Virtual sounds were generated by convolving the original stimuli with binaural room impulse responses (BRIRs). These BRIRs were recorded for each loudspeaker positions using in-ear microphones (Brüel & Kjær type 4101-B) mounted on a head-and-torso simulator (Head Acoustics HMS II.3-33) positioned at the participant's seating location. The BRIRs capture all acoustic characteristics of sound propagation from the loudspeakers to the listener, including reflections, absorption, and timbre, enabling a realistic reproduction of room acoustics (Blauert, 1996; Møller, 1992; Zhang et al., 2017). In addition, a headphone compensation filter was applied which accounts for spectral coloration introduced by the headphones. To this end, their transfer functions were measured multiple times and an inverse filter was derived (Masiero and Fels, 2011). This compensation filter was subsequently applied to the binaural signals to reduce headphone-induced spectral distortions.

### 2.4 Stimuli, task, and procedure

Participants completed a selective spatial attention task using speech stimuli, performed both in an auditory-only condition and in an audio-visual condition where the auditory stimuli were accompanied by visual patterns. In each trial of the task, two simultaneous speech stimulus pairs, presented from two distinct spatial locations, were presented to the participants. Each pair consisted of one of four two-syllable words (Alpha, Bravo, Charlie, Lima; 500 ms each), with one serving as the cue and the other as a distractor, followed by one of eight one-syllable German numerals (Eins [1], Zwei [2], Drei [3], Vier [4], Fünf [5], Sechs [6], Acht [8], Neun [9]), with a comparable duration of approximately 500 ms (ranging from 460 to 498 ms; *M* = 478.50 ms). A silent interval of 300 ms separated the word and the numeral (see Fig. 1C). Each stimulus was followed by a jittered inter-stimulus-interval between 1500 and 1900 ms, resulting in an overall trial length between 2800 and 3200 ms. The combinations of words and numerals were presented in a pseudo-randomized order.

The speech stimuli were generated using an online text-to-speech converter (Voicemaker, 2026), employing a male (Voice ID: ai2-STD-de-DE-Thomas) and a female German synthetic voice (Voice ID: ai2-STD-de-DE-Mona), with fundamental frequencies of 125 Hz and 208 Hz, respectively. Stimuli were subsequently post-processed for loudness normalization and duration adjustments using MATLAB (R2024a; The MathWorks Inc, 2024) and Audacity (v.3.6.4; Audacity-Team, 2024). To avoid acoustic transients, a 10-ms fade-in and fade-out were applied to each word and numeral. The sound pressure level was calibrated to approximately 65 dB_SPL_, measured at 1 m in front of each loudspeaker using a sound level meter (NTi Audio XL2). Calibration was performed using a continuous concatenation of all speech stimuli (words and numerals) without inter-stimulus pauses. Consequently, the reported level reflects the time-averaged A-weighted equivalent continuous sound level (Leq) of the full stimulus sequence. To ensure precise timing of sound onsets at the participants’ ears, regardless of loudspeaker position, the time-of-arrival of the stimuli from each position in the real environment was measured and compensated accordingly.

In the audio-visual condition, the auditory stimuli were accompanied by abstract visual patterns presented at the corresponding spatial locations. More specifically, each target word was associated with a distinct visual pattern consisting of a grey background and a black stripe in a specific orientation (see Fig. 1D). The pattern was displayed only during the target word in the first 500 ms of each trial (see Fig. 1C). Abstract visual stimuli with identical colors and proportions were used to control for semantic processing and object recognition effects, ensuring that observed effects were driven by spatial and attentional mechanisms rather than higher-level visual content.

In each trial, participants were required to evaluate the numeral presented at the cued target location while ignoring the simultaneously presented distractor numeral. More specifically, participants were first required to identify which of the two simultaneously active spatial positions played the instructed target word and then to direct their attention to that position. The spatial position to be attended was indicated by the cue word assigned for the current block (e.g., “Alpha” in the auditory condition; see Fig. 1E) or, in the audio-visual condition, by the cue word accompanied by a simultaneously presented visual pattern. Following this, participants had to evaluate whether the numeral presented at the cued position was odd or even. Participants were instructed to respond as quickly as possible using a joystick – pulling it to one side for even numerals and to the other side for odd numerals. No feedback about correct or false responses was given during the experiment. The cue word varied across blocks, with each of the four words serving as the cue once.

The task comprised a total of 1152 trials, divided into eight blocks of 144 trials each (576 trials per stimulation). Participants could take short breaks between blocks. To ensure task understanding, 20 practice trials were presented at the beginning of the auditory-only and audio-visual conditions. The starting environment (real or virtual), starting stimulation (auditory-only or audio-visual), and the mapping of joystick direction (left/right) to numeral type (even/odd) were counterbalanced across participants. However, to maintain consistency, the order of stimulations, target word assignments, and joystick configurations remained the same for each participant across both the real and virtual environments.

### 2.5 Analyses and data recording

#### 2.5.1 Statistical analyses and data reporting

In the following sections, means (*M*) are reported either as descriptive statistics or as model-based estimated marginal means, depending on the analysis. For preprocessing and data quality measures (e.g., number of rejected epochs), *M* and standard deviations (*SD*) reflect descriptive averages across participants. For inferential analyses (behavioral and neurophysiological models), *M* refers to model-based estimated marginal means (posterior means) derived from the fitted Bayesian model. Uncertainty is quantified using 95% highest posterior density (HPD) intervals. Depending on the reported effect, values are averaged across the remaining experimental factors. Specifically, *M* and *SD* for main effects (environment, stimulation, target-distractor combination) are averaged across the two remaining factors. *M* and *SD* for two-way combinations (e.g., *M*_*real,auditory-only*_) are averaged across the remaining factors (e.g., target-distractor combinations), such that any factor not explicitly included in the notation is collapsed across all its levels.

Participants' responses were classified according to the spatial dimensions of the target and the distractor in each trial. Trials with targets/distractors presented from left or right positions were assigned to the “azimuth” dimension, whereas trials with targets/distractors from near or far positions were assigned to the “depth” dimension. For example, if the target was presented on the left and the distractor on the right, both were coded as belonging to the azimuth dimension. If the target appeared at the near position and the distractor on the left, the target was classified as belonging to the depth dimension and the distractor to the azimuth dimension. This procedure allowed us to analyze the influence of spatial dimension on behavior and neural responses without averaging across individual spatial positions. Moreover, grouping trials by dimension increased statistical power and reduced the complexity of the analyses, facilitating the interpretation of how azimuth and depth differentially affect auditory spatial attention. For the behavioral analyses, accuracy was defined as the proportion of correctly answered trials per environment (real vs. virtual), stimulation (auditory-only vs. audio-visual), and target-distractor combination (azimuth vs. depth). In addition, reaction times were computed as the time interval between stimulus onset and the participant's response.

Behavioral data were analyzed using Bayesian generalized linear mixed models (GLMMs) on trial-level data. Accuracy was modeled using a binomial distribution with a logit link, while reaction times were log-transformed prior to analysis and modeled using a Gaussian distribution with an identity link. Reference levels for the factors were the real environment, auditory-only condition, and the target-distractor combination with both presented in azimuth. To account for repeated measures and participant-specific variability, the model included random intercepts and random slopes for environment, stimulation, and target-distractor combination for each participant. In the results section, we report odds ratios (OR) for behavioral outcomes. The OR quantifies how much more likely a correct response is in one condition compared with a reference condition: OR = 1 indicates no difference, OR > 1 indicates a higher likelihood in the first condition, and OR < 1 indicates a higher likelihood in the reference condition. For example, OR = 2 means that a correct response is twice as likely in the first condition as in the reference condition. Confidence intervals ([a, b]) indicate the range within which the true population parameter is expected to lie with 95% confidence. Post-hoc pairwise comparisons based on estimated marginal means were Bonferroni-corrected to control for multiple comparisons.

Neurophysiological data were analyzed using Bayesian repeated measures ANOVAs with the within-subject factors environment (real vs. virtual), stimulation (auditory-only vs. audio-visual), and target-distractor combination. Post-hoc tests were conducted to follow up significant main and interaction effects. Here, Bayes factors (*BF*) are reported as evidence measures for individual comparisons.

We chose GLMMs for behavioral measures to appropriately model their trial-wise and bounded nature, and ANOVAs for ERP amplitudes based on condition-wise averaged waveforms per participant to remain consistent with standard electrophysiological analysis practices. We used Bayes factor analysis, as it directly quantifies how much more likely the observed data are under the alternative hypothesis (H1) than under the null hypothesis (H0) (Kass and Raftery, 1995). Unlike *p*-values, *BF*s provide a graded measure of evidence, allowing assessment of support for both H1 (>1) and H0 (<1), with a value of 1 indicating no preference. *BF*s can be interpreted along a continuum from anecdotal to extreme evidence, offering a nuanced evaluation of experimental effects (Jeffreys, 1961). All statistical analyses were carried out using R (v.4.5.1) in RStudio (v.2025.05.1; Posit Team, 2025) and JASP (v.0.95; JASP Team, 2025). All analyses were based on repeated within-subject measurements, with each participant contributing multiple observations per condition across environment, stimulation, and target-distractor combination. Behavioral analyses were conducted at the trial level, whereas ERP analyses were based on condition-wise averages across trials within each participant.

#### 2.5.2 Drift-diffusion modeling

To further investigate the cognitive processes underlying participants’ decisions, a hierarchical drift-diffusion model (DDM) was used. The DDM is a well-established computational framework for analyzing reaction times and choice accuracy in two-alternative forced-choice tasks (Ratcliff, 1978; Ratcliff et al., 2016). It conceptualizes decision-making as a process of noisy evidence accumulation: Over time, sensory or cognitive information is integrated until it reaches one of two decision boundaries, which triggers a response. A key parameter in the model is the drift rate (*μ*), representing the average speed at which evidence accumulates in favor of one choice, with higher values indicating faster and more efficient information processing. Other important parameters include the boundary separation (*bs*), reflecting response caution and how much evidence is required before making a decision, the non-decision time (*ndt*), capturing processes unrelated to the decision itself, such as sensory encoding and motor execution, and the starting point (bias), indicating any predisposition toward one choice before evidence accumulation begins. In the present study, the model included fixed effects of environment, stimulation, and target-distractor combination, as well as their interactions, with a random intercept for participants. The bias parameter was fixed at 0.5 to reflect an unbiased decision onset (odd or even numeral).

#### 2.5.3 EEG preprocessing and ERP quantification

Continuous EEG activity was recorded from 64 Ag/AgCl electrodes (EasyCap, Wörthsee, Germany) according to the international 10-20 system. The signals were acquired with a sampling rate of 1000 Hz using a QuickAmp DC amplifier (BrainVision, Gilching, Germany), and electrode impedances maintained below 15 kΩ. FCz served as the reference and AFz as the ground electrode. The HMD was placed directly on the EEG cap, with thin custom-made spacers positioned beneath the head strap to prevent contact with the central midline electrodes and ensure stable recordings. EEG data was preprocessed offline using the EEGLAB (v2025.0.0; Delorme and Makeig, 2004) and ERPLAB (v12.01; Lopez-Calderon and Luck, 2014) toolboxes in MATLAB, along with custom code. Preprocessing was performed separately for each participant and environment. The raw data were filtered with cutoff frequencies of 0.1 Hz (high-pass) and 30 Hz (low-pass). Channels corresponding to bad electrodes (e.g., those with excessive noise or flat lines) were removed and interpolated using spherical interpolation. Data was then re-referenced to the common average reference (CAR), i.e., the average signal across all 64 electrodes. Further data cleaning was performed using independent component analysis (ICA). In preparation for this, a subset of the EEG data was generated with cut-off frequencies of 1 and 30 Hz, down-sampled to 200 Hz for faster computation, and only every second trial was included. The continuous EEG signal was segmented into stimulus-locked epochs of 2200 ms, ranging from −200 ms to 2000 ms relative to stimulus onset. The interval from −200 ms to stimulus onset served as the pre-stimulus baseline. An initial automatic artifact rejection based on amplitude threshold and a statistical probability criterion set at five standard deviations, with a maximum of five epochs rejected per component, resulted in *M*_*real,auditory-only*_ = 15.63 (*SD*_*real,auditory-only*_ = 9.99) removed epochs per participant in the real environment and auditory-only condition, *M*_*real,audio-visual*_ = 10.25 (*SD*_*real,audio-visual*_ = 9.08) removed epochs per participant in the real environment and audio-visual condition, *M*_*virtual,auditory-only*_ = 11.75 (*SD*_*virtual,auditory-only*_ = 8.74) removed epochs for the auditory-only condition in the virtual environment, and *M*_*virtual,audio-visual*_ = 10.46 (*SD*_*virtual,audio-visual*_ = 15.33) removed epochs for the audio-visual condition in the virtual environment. Afterwards, the ICA was performed, followed by the application of the ICLabel algorithm (v1.6, Pion-Tonachini et al., 2019). The resulting component classifications and ICA weights were then transferred back to the original EEG data. Components were excluded if their probability was below 30% for the brain category or above 30% for eye, activity. On average, the number of removed components per participant was as follows: auditory-only condition in the real environment: *M*_*real,auditory-only*_ = 26.88 (*SD*_*real,auditory-only*_ = 7.65), audio-visual condition in the real environment: *M*_*real,audio-visual*_ = 30.54 (*SD*_*real,audio-visual*_ = 9.25), auditory-only condition in the virtual environment: *M*_*virtual,auditory-only*_ = 30.33 (*SD*_*virtual,auditory-only*_ = 7.01) and audio-visual condition in the virtual environment: *M*_*virtual,audio-visual*_ = 32.17 (*SD*_*virtual,audio-visual*_ = 7.17). Remaining artifactual and noisy epochs were removed by applying amplitude thresholding. Here, the average number of rejected epochs per participant was as follows: *M*_*real,auditory-only*_ = 5.58 (*SD*_*real,auditory-only*_ = 6.69), *M*_*real,audio-visual*_ = 6.25 (*SD*_*real,audio-visual*_ = 14.15), *M*_*virtual,auditory-only*_ = 1.88 (*SD*_*virtual,auditory-only*_ = 4.15), *M*_*virtual,audio-visual*_ = 3.92 (*SD*_*virtual,audio-visual*_ = 12.61).

For quantifying ERP amplitudes, we used the MATLAB function developed by Liesefeld (2018). The P1, N1, and P2 components were identified for each environment, stimulation, and target-distractor combination per participant by extracting peak amplitudes within predefined latency windows: 50-100 ms for P1, 50-200 ms for N1, and 100-400 ms for P2. The CNV was quantified as the mean amplitude in the 500-800 ms interval following the onset of the cue, corresponding to the silent gap between the cue and the subsequent numeral. Based on previous literature (e.g., Friedman et al., 1993; Kononowicz and Penney, 2016; Näätänen and Picton, 1987), and observed topographical patterns (see 3.2), all components were derived from averages across six fronto-central electrodes (Cz, C1, C2, FCz, FC1, FC2) to characterize ERP responses.

## 3 Results

### 3.1 Behavioral results

#### 3.1.1 Accuracy

**Fig. 2:**
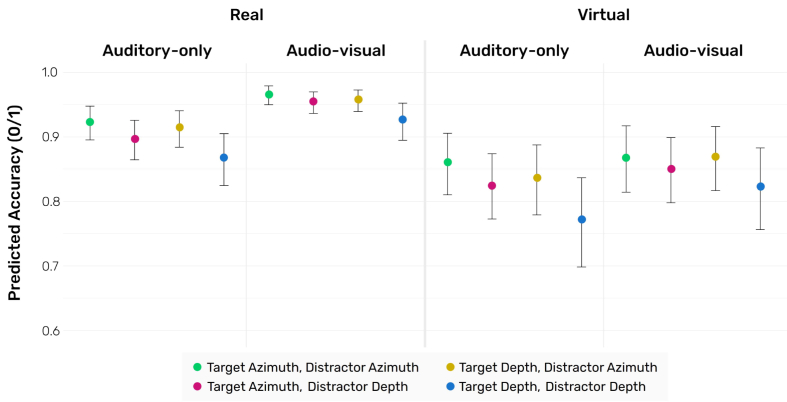
Predicted accuracy for correctly classifying the numeral at the target position as odd or even, shown separately for each environment, stimulation, and target-distractor combination. Error bars represent 95% highest posterior density (HPD) intervals.

The results for accuracy, including all main and interaction effects, are summarized in Table 1. Further, Fig. 2 shows the predicted accuracy across environment, stimulation, and target-distractor combination. Overall, accuracy was high but varied systematically. The model-predicted accuracy (expressed as a proportion) was higher in the real environment (*M*_*real*_ = 0.93 [0.91, 0.95]) than in the virtual environment (*M*_*virtual*_ = 0.84 [0.79, 0.89]). A pairwise comparison indicated that the odds of a correct response were significantly higher in the real environment compared with the virtual environment (OR = 2.62 [1.75, 3.75]). Further, accuracy was generally higher in the audio-visual condition (*M*_*audio-visual*_ = 0.92 [0.89, 0.94]) than in the auditory-only condition (*M*_*auditory-only*_ = 0.87 [0.83, 0.90]), with significantly lower odds of a correct response in the auditory-only condition (OR = 0.61 [0.50, 0.74]). Moreover, accuracy varied across target-distractor combinations, being highest when both stimuli were presented in azimuth (*M*_*azimuth, azimuth*_ = 0.92 [0.89, 0.94]), followed by target in depth and distractor in azimuth (*M*_*depth, azimuth*_ = 0.91 [0.88, 0.93]), target in azimuth and distractor in depth (*M*_*azimuth, depth*_ = 0.89 [0.86, 0.92]), both target and distractor in depth (*M*_*depth, depth*_ = 0.86 [0.82, 0.90]). Pairwise comparisons confirmed that performance was highest when both stimuli were in azimuth and lowest when both were in depth (all OR > 1, see Table 2).

**Table 1:** Posterior estimates for the accuracy model. Values reflect the effect of each predictor on the log-odds of a correct response relative to the reference levels. The intercept represents the baseline log-odds in the reference condition (environment = real, stimulation = auditory, target-distractor combination = azimuth/azimuth). Positive values indicate higher odds of a correct response, whereas negative values indicate lower odds. Main effects and interactions showing substantial posterior evidence (95% highest posterior density (HPD) intervals excluding zero) are indicated in bold.

Predictor	Median	SE	95% HPD
Intercept	2.49	0.19	[2.12, 2.87]

**Environment Virtual**	**−0.66**	**0.21**	**[-1.07, -0.25]**

**Stimulation Audio-visual**	**0.86**	**0.16**	**[0.55, 1.16]**

**Target Azimuth, Distractor Depth**	**−0.32**	**0.10**	**[-0.52, -0.13]**
Target Depth, Distractor Azimuth	−0.11	0.09	[-0.29, 0.07]
**Target Depth, Distractor Depth**	**−0.60**	**0.10**	**[-0.79, -0.41]**

**Virtual × Audio-visual**	**−0.80**	**0.15**	**[-1.08, -0.50]**

Virtual × Target Azimuth, Distractor Depth	0.05	0.11	[-0.16, 0.27]
Virtual × Target Depth, Distractor Azimuth	−0.08	0.11	[-0.30, 0.14]
Virtual × Target Depth, Distractor Depth	−0.00	0.12	[-0.24, 0.24]

Audio-visual × Target Azimuth, Distractor Depth	0.03	0.14	[-0.26, 0.31]
Audio-visual × Target Depth, Distractor Azimuth	−0.11	0.15	[-0.39, 0.18]
Audio-visual × Target Depth, Distractor Depth	−0.21	0.15	[-0.50, 0.10]

Virtual × Audio-visual × Target Azimuth, Distractor Depth	0.10	0.18	[-0.25, 0.44]
Virtual × Audio-visual × Target Depth, Distractor Azimuth	0.31	0.18	[-0.04, 0.66]
**Virtual × Audio-visual × Target Depth, Distractor Depth**	**0.46**	**0.19**	**[0.08, 0.83]**

**Table 2:** Post-hoc pairwise comparisons of accuracy for the main effect of target-distractor combinations, the interaction between environment and stimulation, and the three-way interaction. Values are odds ratios (ORs) with 95% highest posterior density (HPD) intervals. Each OR compares the row condition (first column) with the column condition (top header). ORs >1 indicate higher accuracy in the row condition relative to the column condition. ORs <1 indicate lower accuracy.

Target-distractor combination	Azimuth, Depth	Depth, Azimuth	Depth, Depth
Azimuth, Azimuth	1.29 [1.14, 1.47]	1.13 [1.03, 1.24]	1.80 [1.59, 2.01]
Azimuth, Depth		0.88 [0.77, 0.98]	1.39 [1.21, 1.56]
Depth, Azimuth			1.59 [1.44, 1.76]

**Environment × Stimulation**	**Virtual Auditory-only**	**Real Audio-visual**	**Virtual Audio-visual**

Real Auditory-only	1.96 [1.29, 2.78]	0.46 [0.36, 0.56]	1.59 [1.00, 2.33]
Virtual Auditory-only		0.23 [0.14, 0.34]	0.81 [0.66, 0.99]
Real Audio-visual			3.49 [2.27, 4.98]

**Environment × Stimulation with both stimuli in depth**	**Virtual Auditory-only**	**Real Audio-visual**	**Virtual Audio-visual**

Real Auditory-only	1.95 [1.22, 2.82]	0.52 [0.39, 0.67]	1.42 [0.85, 2.10]
Virtual Auditory-only		0.27 [0.16, 0.41]	0.73 [0.57, 0.92]
Real Audio-visual			2.72 [1.65, 3.95]

A significant interaction between environment and stimulation indicated that the audio-visual benefit differed between environments. In the real environment, accuracy increased substantially from the auditory-only (*M*_*real,auditory-only*_ = 0.90 [0.87, 0.93]) to the audio-visual condition (*M*_*real,audio-visual*_ = 0.95 [0.93, 0.97]), whereas in the virtual environment, the improvement was smaller (*M*_*virtual,auditory-only*_ = 0.83 [0.77, 0.88]; *M*_*virtual,audio-visual*_ = 0.85 [0.80, 0.91]). Pairwise comparisons revealed that the odds of a correct response were significantly higher in the auditory-only condition in the real environment compared with the auditory-only condition in the virtual environment (see Table 2). Moreover, accuracy in the audio-visual condition in the real environment exceeded that in the virtual environment. Comparisons within environments further indicated a significant audio-visual benefit in the real environment and a smaller, though still present, improvement in the virtual environment.

Furthermore, the significant three-way interaction indicated that the audio-visual benefit differed between environments depending on the spatial configuration of the stimuli. This effect was particularly pronounced when both target and distractor were presented in the depth dimension. Here, accuracy increased from *M*_*real,auditory-only*_ = 0.87 [0.82, 0.91] to *M*_*real,audio-visual*_ = 0.93 [0.90, 0.95], whereas in the virtual environment, performance increased from *M*_*virtual,auditory-only*_ = 0.77 [0.70, 0.84] to *M*_*virtual,audio-visual*_ = 0.82 [0.76, 0.88]. Pairwise contrasts confirmed that this audio-visual benefit was significantly larger in the real than in the virtual environment (OR = 2.72 [1.65, 3.95]; see Table 2).

#### 3.1.2 Reaction times

**Fig. 3:**
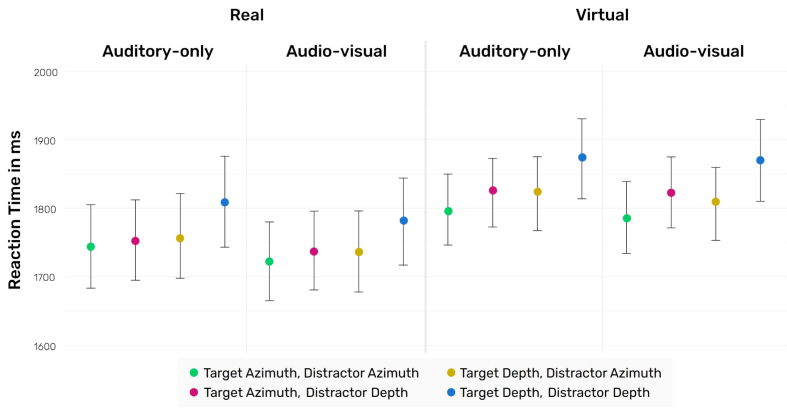
Estimated marginal reaction times (posterior medians) shown separately for each environment, stimulation, and target-distractor combination. Error bars represent 95% highest posterior density (HPD) intervals. Values were back-transformed from the log scale to milliseconds for interpretability. HPD intervals may be asymmetrical after back-transformation.

Reaction time results, including all main effects and interactions, are summarized in Table 3. Fig. 3 shows the model-predicted reaction times across environment, stimulation, and target-distractor combination. The analysis revealed a main effect of environment with slightly longer reaction times in the virtual environment (*M*_*virtual*_ = 1825.57 ms [1770.97, 1873.09]) compared with the real environment (*M*_*real*_ = 1754.10 ms [1696.55, 1813.06]). The contrast between the virtual and the real corresponds to a ratio of 1.04 [1.02, 1.06], indicating that reaction times in the virtual environment were 4% longer than in real environment.

**Table 3:** Posterior estimates for the reaction time model. Values reflect the effect of each predictor on reaction time (ms) relative to the reference levels. The intercept represents the baseline reaction time in the reference condition (environment = real, stimulation = auditory, target-distractor combination = azimuth/azimuth). Positive values indicate slower responses, whereas negative values indicate faster responses. Main effects and interactions showing substantial posterior evidence (95% highest posterior density (HPD) intervals excluding zero) are indicated in bold. Point estimates correspond to posterior medians.

Predictor	Median	SE	95% HPD
Intercept	7.46	0.02	[7.43, 7.50]

**Environment Virtual**	**0.03**	**0.01**	**[0.01, 0.05]**

Stimulation Audio-visual	−0.01	0.01	[-0.03, 0.00]

Target Azimuth, Distractor Depth	0.00	0.00	[-0.00, 0.01]
Target Depth, Distractor Azimuth	0.01	0.00	[-0.00, 0.02]
**Target Depth, Distractor Depth**	**0.04**	**0.01**	**[0.03, 0.05]**

Virtual × Audio-visual	0.01	0.01	[-0.01, 0.02]

Virtual × Target Azimuth, Distractor Depth	0.01	0.01	[0.00, 0.02]
Virtual × Target Depth, Distractor Azimuth	0.01	0.01	[-0.00, 0.02]
Virtual × Target Depth, Distractor Depth	0.01	0.01	[-0.01, 0.02]

Audio-visual × Target Azimuth, Distractor Depth	0.00	0.01	[-0.01, 0.01]
Audio-visual × Target Depth, Distractor Azimuth	0.00	0.01	[-0.01, 0.01]
Audio-visual × Target Depth, Distractor Depth	0.00	0.01	[-0.01, 0.01]

Virtual × Audio-visual × Target Azimuth, Distractor Depth	0.00	0.01	[-0.01, 0.02]
Virtual × Audio-visual × Target Depth, Distractor Azimuth	0.00	0.01	[-0.02, 0.01]
Virtual × Audio-visual × Target Depth, Distractor Depth	0.01	0.01	[-0.01, 0.02]

Furthermore, reaction times varied across target-distractor combinations, with responses being longest when both stimuli were presented in depth (*M*_*depth, depth*_ = 1832.60 ms [1772.29, 1887.71]). The other combinations – target in azimuth and distractor in depth (*M*_*azimuth, depth*_ = 1783.34 ms [1735.32, 1836.66]), target in depth and distractor in azimuth (*M*_*depth, azimuth*_ = 1780.77 ms [1728.49, 1834.78]), and both stimuli in azimuth (*M*_*azimuth, azimuth*_ = 1761.11 ms, [1710.45, 1812.68]) – differed only slightly from each other. Pairwise comparisons indicated that all combinations involving an azimuth position yielded credibly shorter reaction times than the combination with both stimuli in depth. Specifically, reaction times were about 4% shorter when both stimuli were in azimuth and about 3% shorter when one stimulus was in azimuth and the other in depth (see Table 4 for all pairwise comparisons). No interaction effects were observed.

**Table 4:** Pairwise post-hoc comparisons of reaction times across target-distractor combinations. Values represent estimated reaction time ratios (first condition ÷ second condition) derived from the Bayesian GLMM, together with 95% highest posterior density (HPD) intervals. Each ratio compares the row condition (first column) with the column condition (top header). Ratios <1 indicate that the first combination resulted in faster responses than the second, whereas ratios >1 indicate slower responses.

Target-distractor combination	Azimuth, Depth	Depth, Azimuth	Depth, Depth
Azimuth, Azimuth	0.99 [0.98, 0.99]	0.99 [0.98, 1.00]	0.96 [0.95, 0.97]
Azimuth, Depth		1.00 [1.00, 1.01]	0.97 [0.97, 0.98]
Depth, Azimuth			0.97 [0.97, 0.98]

#### 3.1.3 Drift-diffusion model

A hierarchical DDM was fitted using the formula

Reaction Time | Decision ∼ Environment * Stimulation *

Target-Distractor Combination + (1 | Participant)

to examine how environment, stimulation, and target-distractor combination influenced evidence accumulation, including a random intercept for participants. The bias parameter was fixed at 0.5 to reflect an unbiased decision onset (odd vs. even numeral).

Random effects for participants indicated moderate variability in baseline drift rates relative to the overall mean (*SD* = 0.42 [0.32, 0.57]), reflecting inter-individual differences in evidence accumulation. Participants’ boundary separation (*bs* = 2.33) and non-decision time (*ndt* = 0.35 s) were stable across environments, stimulations, and target-distractor combinations, indicating consistent response caution and sensory-motor processing. Table 5 summarizes drift rates (*μ*) for each environment, stimulation, and target-distractor combination, as well as their interactions, along with corresponding *BF*s. Drift rates were slower in the virtual compared with the real environment (*μ* = −0.28, *BF*_*10*_ = 6.00 × 10^5^) and slightly faster in the audio-visual compared with the auditory-only condition (*μ* = 0.17, *BF*_*10*_ = 51.46). Regarding target-distractor combinations, decisions were fastest when both target and distractor were presented in azimuth (used as reference), moderately slower for mixed-dimension trials (target in azimuth, distractor in depth: *μ* = −0.13, *BF*_*10*_ = 38.36; target in depth, distractor in azimuth: *μ* = −0.08, *BF*_*10*_ = 0.59), and slowest when both were presented in the depth dimension (*μ* = −0.39, *BF*_*10*_ = 1.26 × 10^10^).

**Table 5:** Drift rates (μ) estimated from the hierarchical drift-diffusion model. Positive values indicate faster evidence accumulation toward the correct response, whereas negative values indicate slower or less efficient accumulation. The intercept represents the baseline drift rate in the reference condition (environment = real, stimulation = auditory-only, target-distractor combination = azimuth/azimuth). All other values represent deviations from this baseline for main effects and interactions. Main effects and interactions with substantial evidence are indicated in bold.

Predictor	Drift rate (*μ*)	95% HPD	*BF*_*10*_
Intercept (Baseline drift rate)	1.74	[1.55, 1.92]	

**Environment Virtual**	**−0.28**	**[-0.35, -0.21]**	**6.00×10^5^**

**Stimulation Audio-visual**	**0.17**	**[0.09, 0.25]**	**51.46**

**Target Azimuth, Distractor Depth**	**−0.13**	**[-0.20, -0.07]**	**38.36**
Target Depth, Distractor Azimuth	−0.08	[-0.14, −0.01]	0.587
**Target Depth, Distractor Depth**	**−0.39**	**[-0.47, -0.32]**	**1.26×10^10^**

**Virtual** × **Audio-visual**	**−0.16**	**[-0.27, -0.06]**	**3.96**

Virtual × Target Azimuth, Distractor Depth	−0.05	[-0.15, 0.04]	0.091
Virtual × Target Depth, Distractor Azimuth	−0.09	[-0.18, −0.00]	0.372
Virtual × Target Depth, Distractor Depth	−0.09	[-0.20, 0.01]	0.279

Audio-visual × Target Azimuth, Distractor Depth	0.05	[-0.04, 0.14]	0.075
Audio-visual × Target Depth, Distractor Azimuth	0.01	[-0.08, 0.10]	0.051
Audio-visual × Target Depth, Distractor Depth	0.07	[-0.03, 0.18]	0.125

Virtual × Audio-visual × Target Azimuth, Distractor Depth	−0.01	[-0.14, 0.12]	0.068
Virtual × Audio-visual × Target Depth, Distractor Azimuth	0.11	[-0.02, 0.24]	0.253
Virtual × Audio-visual × Target Depth, Distractor Depth	0.10	[-0.05, 0.24]	0.168

Regarding interaction effects, only the combination of the virtual environment and the audio-visual condition resulted in a modest decrease in drift rate (*μ* = −0.16, *BF*_*10*_ = 3.96), while all other interactions showed little evidence for modulation (*BF*_*10*_ < 0.40). Overall, slower responses in the virtual environment and in only-depth trials were primarily driven by reduced drift rates, whereas boundary separation and non-decision processes remained largely constant.

### 3.2 Event-related potentials

**Fig. 4:**
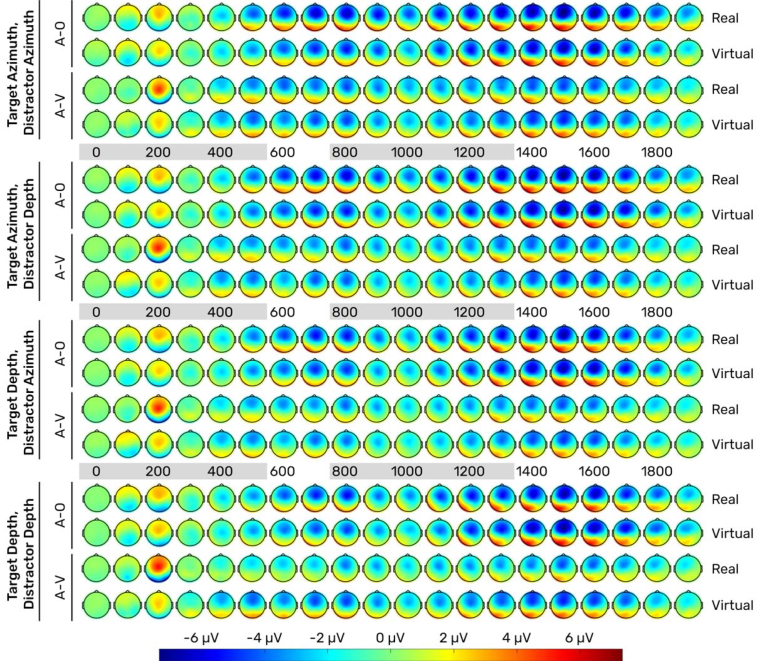
Averaged EEG topographies across the 2000 ms following cue onset, encompassing the early P1, N1, P2, and CNV components. Topographies are shown separately for each target-distractor combination, stimulation (A-O = auditory-only, A-V = audio-visual), and environment. The time windows in which the stimuli (word and numeral) occurred are highlighted in grey.

Fig. 4 illustrates the averaged EEG topographies for 2000 ms following the onset of the cue, displayed in intervals of 100 ms across environment, stimulation, and target-distractor combination. Visual inspection revealed a prominent fronto-central positive deflection around 200 ms in all environments, stimulations, and target-distractor combinations, reflecting the P2, followed by a rising fronto-central negativity starting approximately 400-500 ms after onset of the first stimuli. This negativity reached its peak around 800 ms (reflecting the CNV), decreased for 200-300 ms, and then increased again before gradually returning to baseline toward the end of the 2000 ms window. Overall, these patterns support the use of a fronto-central electrode cluster for the subsequent ERP analyses.

**Fig. 5:**
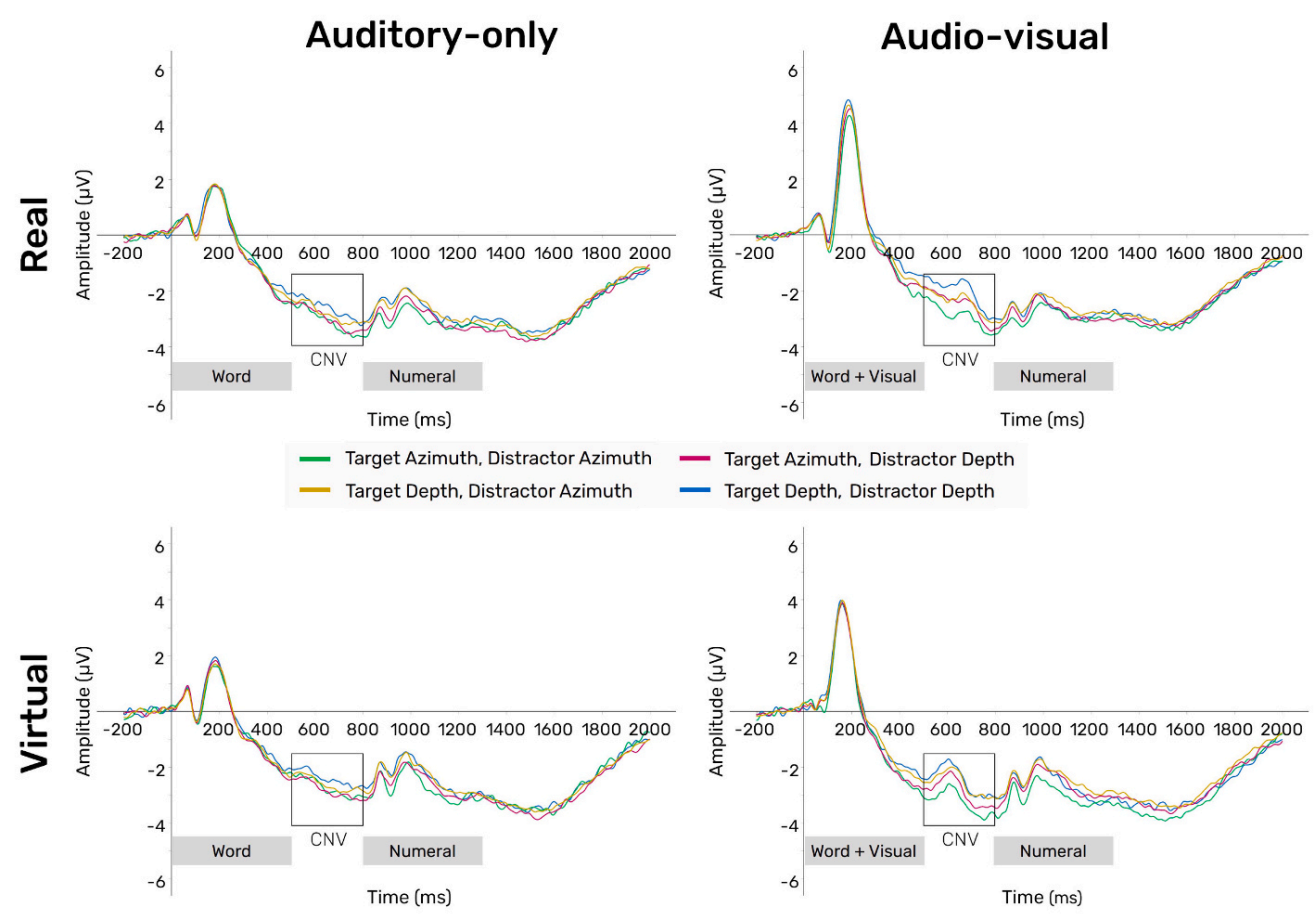
Grand-averaged ERP waveforms recorded in the real (top) and virtual (bottom) environments as well as in the auditory-only (left panel) and audio-visual (right panel) condition averaged over fronto-central electrodes (Cz, C1, C2, FCz, FC1, FC2). Grey-filled boxes indicate the temporal windows of the cue and the numeral. The CNV mean amplitude was measured in the interval between these two events, as indicated by the black-lined box.

Fig. 5 provides a visual comparison of the grand-averaged ERP waveforms for each environment, stimulation, and target-distractor combination at fronto-central electrodes, including the P1-N1-P2 complex and the CNV. In the following, the effects of environment, stimulation, and target-distractor combination, as well as their interactions, on ERP amplitudes are described.

#### 3.2.1 P1-N1-P2

To explore the effects of environment, stimulation, and target-distractor combinations on the peak amplitudes of the early event-related potentials, Bayesian repeated-measures ANOVAs were conducted.

For the P1, the null model provided the best account of the data (*BF*_*M*_ = 13.18, *P(M|data)* = 0.423). Models including main effects of environment (*BF*_*M*_ = 5.44, *P(M|data)* = 0.232), stimulation (*BF*_*M*_ = 2.27, *P(M|data)* = 0.112), or target-distractor combination (*BF*_*M*_ = 1.30, *P(M|data)* = 0.067) were less supported. Models including interaction effects received very low support (all *BF*_*M*_ < 1, *P(M|data)* ≤ 0.067). Inclusion BFs further indicated the absence of meaningful effects (environment: *BF*_*incl*_ = 0.21; stimulation: *BF*_*incl*_ = 0.11; target-distractor combination: *BF*_*incl*_ = 0.06; all interactions *BF*_*incl*_ ≤ 0.04). Overall, these results show that P1 amplitude was not reliably modulated by environment, stimulation, target-distractor combination, or any of their interactions.

For N1 peak amplitude, also the null model provided the best account of the data (*BF*_*M*_ = 8.66, *P(M|data)* = 0.325). Models including only environment (*BF*_*M*_ = 2.01, *P(M|data)* = 0.100), only stimulation (*BF*_*M*_ = 2.99, *P(M|data)* = 0.143), or both factors and their interaction (*BF*_*M*_ = 3.26, *P(M|data)* = 0.153) received only anecdotal support, while models including only combinations (*BF*_*M*_ = 1.46, *P(M|data)* = 0.075) or two- and higher-order interactions were generally unsupported (all *BF*_*M*_ ≤ 1.33, *P(M|data)* ≤ 0.069). Inclusion BFs indicated weak evidence for main effects of environment (*BF*_*incl*_ = 0.27), stimulation (*BF*_*incl*_ = 0.32), and their interaction (*BF*_*incl*_ = 0.72), whereas target-distractor combinations (*BF*_*incl*_ = 0.13) and further interactions were not supported (all *BF*_*incl*_ ≤ 0.29, three-way *BF*_*incl*_ = 2.23 × 10^−5^). Overall, these results suggest that similar to P1, N1 amplitude was largely unaffected by environment, stimulation, target-distractor combinations, or their interactions.

For P2 peak amplitude, the model including environment, stimulation, target-distractor combinations, and the interaction between environment and stimulation was best supported (*BF*_*M*_ = 19.74, *P(M|data)* = 0.523). The second-best fitting model, only receiving anecdotal support, included only the main effects of environment, stimulations, and target-distractor combinations (*BF*_*M*_ = 2.94, *P(M|data)* = 0.140). All other models showed *BF*_*M*_ ≤ 1.18 and *P(M|data)* ≤ 0.108. Inclusion *BF*s indicated strong support for main effects of environment (*BF*_*incl*_ = 3.78), stimulation (*BF*_*incl*_ = 1.12 × 10^6^), and the interaction between environment and stimulation (*BF*_*incl*_ = 5.27), and moderate support for target-distractor combinations (*BF*_*incl*_ = 1.85). All interactions involving the target-distractor combinations showed no support (all *BF*_*incl*_ ≤ 0.198).

Post-hoc Bayesian t-tests revealed larger P2 amplitudes in the real than in the virtual environment (*M*_*real*_ = 3.50 μV, *SD*_*real*_ = 2.38 vs. *M*_*virtual*_ = 3.13 μV, *SD*_*virtual*_ = 1.85; *BF*_*10*_ = 98.82). Furthermore, P2 was higher in the audio-visual compared with the auditory-only condition (*M*_*audio-visual*_ = 4.53 μV, *SD*_*audio-visual*_ = 2.09 vs. *M*_*auditory-only*_ = 2.10 μV, *SD*_*auditory-only*_ = 1.35; *BF*_*10*_ = 1.74 × 10^43^). Although differences between conditions appear subtle in the grand-average ERPs (Fig. 5), quantitative comparisons across target-distractor combinations revealed consistent evidence for higher P2 amplitudes when both target and distractor were presented in the depth dimension, whereas all other comparisons showed no evidence for differences (Table 6).

**Table 6:** Post-hoc Bayesian pairwise comparisons across target-distractor combinations for the P2 and CNV amplitudes. Differences with substantial evidence are indicated in bold.

ERP	Comparison of target-distractor combination		*M*_*1*_*(SD*_*1*_*)*	*M*_*2*_*(SD*_*2*_*)*	*BF*_*10*_
**P2**	Target Depth, Distractor Depth	Target Azimuth, Distractor Azimuth	3.52 (2.29)	3.19 (1.97)	**5.71**
		Target Azimuth, Distractor Depth	3.52 (2.29)	3.26 (2.11)	**27.43**
		Target Depth, Distractor Azimuth	3.52 (2.29)	3.28 (2.11)	**6.91**
	Target Azimuth, Distractor Azimuth	Target Azimuth, Distractor Depth	3.19 (1.97)	3.26 (2.11)	0.149
		Target Depth, Distractor Azimuth	3.19 (1.97)	3.28 (2.11)	0.198
	Target Azimuth, Distractor Depth	Target Depth, Distractor Azimuth	3.26 (2.11)	3.28 (2.11)	0.132
**CNV**	Target Depth, Distractor Depth	Target Azimuth, Distractor Azimuth	−2.36 (1.50)	−2.98 (1.59)	**3188.03**
		Target Azimuth, Distractor Depth	−2.36 (1.50)	−2.77 (1.32)	**1042.60**
		Target Depth, Distractor Azimuth	−2.36 (1.50)	−2.59 (1.23)	**6.24**
	Target Azimuth, Distractor Azimuth	Target Azimuth, Distractor Depth	−2.98 (1.59)	−2.77 (1.32)	**1.45**
		Target Depth, Distractor Azimuth	−2.98 (1.59)	−2.59 (1.23)	**132.40**
	Target Azimuth, Distractor Depth	Target Depth, Distractor Azimuth	−2.77 (1.32)	−2.59 (1.23)	**7.37**

To further investigate the interaction between environment and stimulation, and to assess how environment affected the audio-visual benefit, peak amplitudes were averaged across target-distractor combinations and compared between auditory-only and audio-visual trials. In the real environment, the mean peak amplitude was *M*_*real,audio-visual*_ = 4.90 μV (*SD*_*real,audio-visual*_ = 2.35) for audio-visual trials and *M*_*real,auditory-only*_ = 2.10 μV (*SD*_*real,auditory-only*_ = 1.35) for auditory-only trials. In the virtual environment, peak amplitudes were *M*_*virtual,audio-visual*_ = 4.15 μV (*SD*_*virtual,audio-visual*_ = 1.71) for audio-visual trials and *M*_*virtual,auditory-only*_ = 2.10 μV (*SD*_*virtual,auditory-only*_ = 1.35) for auditory-only trials. Bayesian t-tests on the resulting differences showed that P2 amplitudes were enhanced by the presence of visual cues, with the increase slightly larger in the real than in the virtual environment (*ΔM*_*Real*_ = 2.80, *ΔM*_*Virtual*_ = 2.06, *BF*_*10*_ = 2.22).

#### 3.2.2 CNV

As with early potentials, Bayesian repeated-measures ANOVAs were conducted to examine the effects of the experimental factors on the mean amplitude of the CNV. Here, the model including environment, stimulation, target-distractor combinations, and the interaction between environment and stimulation provided the best account of the data (*BF*_*M*_ = 24.61, *P(M|data)* = 0.578). Models including target-distractor combinations alone (*BF*_*M*_ = 6.67, *P(M|data)* = 0.270) also received some support, whereas all other models received substantially less support (all *BF*_*M*_ ≤ 1.85, all *P(M|data)* ≤ 0.093). Inclusion BFs indicated strong evidence for a main effect of target-distractor combinations (*BF*_*incl*_ = 4753.82, *P(incl|data)* = 1.00), whereas environment (*BF*_*incl*_ = 0.80, *P(incl|data)* = 0.692) and stimulation (*BF*_*incl*_ = 0.61, *P(incl|data)* = 0.631) showed no clear main effects. The interaction between environment and stimulation showed moderate support (*BF*_*incl*_ = 3.09, *P(incl|data)* = 0.588). Overall, these results suggest that the CNV amplitude is primarily driven by the target-distractor combinations, with minimal contributions from environment and stimulation.

Post-hoc Bayesian t-tests showed no evidence for a difference in CNV amplitudes between the real and virtual environments (*M*_*real*_ = −2.66 μV, *SD*_*real*_ = 1.47 vs. *M*_*virtual*_ = −2.69 μV, *SD*_*virtual*_ = 1.39; *BF*_*10*_ = 0.09). Furthermore, there was also no evidence for a difference in CNV amplitude between the audio-visual and auditory-only conditions (*M*_*audio-visual*_ = −2.63 μV, *SD*_*audio-visual*_ = 1.48 vs. *M*_*auditory-only*_ = −2.72 μV, *SD*_*auditory-only*_ = 1.37; *BF*_*10*_ = 0.12). Comparisons across target-distractor combinations indicated differences in CNV amplitudes across all contrasts. Notably, CNV amplitudes were generally highest when both the target and distractor were in azimuth and lowest when both were in depth (see Table 6).

With regard to the interaction between environment and stimulation, the CNV amplitudes were slightly smaller (less negative) for audio-visual trials (*M*_*real,audio-visual*_ = −2.46 μV, *SD*_*real,audio-visual*_ = 1.47) than for auditory-only trials (*M*_*real,auditory-only*_ = −2.85 μV, *SD*_*real,auditory-only*_ = 1.44) in the real environment, whereas in the virtual environment, CNV amplitudes were slightly higher (more negative) for audio-visual trials (*M*_*virtual,audio-visual*_ = −2.80 μV, *SD*_*virtual,audio-visual*_ = 1.47) compared with auditory-only trials (*M*_*virtual,auditory-only*_ = −2.58 μV, *SD*_*virtual,auditory-only*_ = 1.30). Bayesian t-tests on the resulting differences provided anecdotal evidence for a modestly larger audio-visual benefit in the real environment (*ΔM*_*Real*_ = 0.39, *ΔM*_*Virtual*_ = −0.22, BF_10_ = 2.38).

### 3.3 Cross-environment relationships

**Fig. 6:**
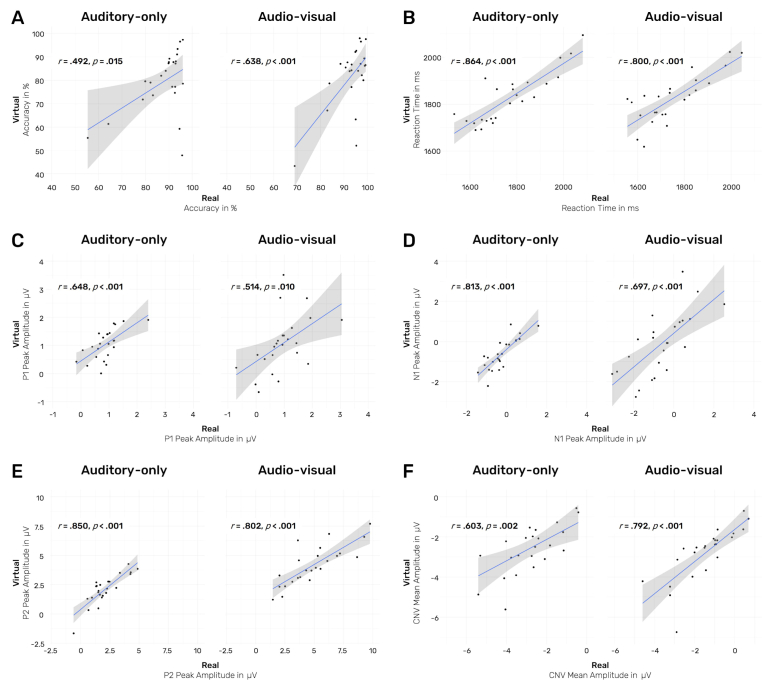
Scatter plots illustrating the relationship between real and virtual environments across (A) accuracy and (B) reaction times as well as (C) P1, (D) N1, (E) P2, and (F) CNV amplitudes, separately for auditory-only and audio-visual stimulation. For each participant, values were averaged across target-distractor combinations before calculating the correlations. Within each panel, individual participant mean values are plotted, where each point represents a single participant. Blue lines indicate linear regression fits with 95% confidence intervals (shaded grey areas). Pearson correlation coefficients (*r*) are shown for each analysis.

To examine the correspondence between measures obtained in the real and virtual environments, Pearson correlations were calculated separately for the auditory-only and audio-visual conditions using participants’ values averaged across target-distractor combinations. Significant positive correlations were observed for all behavioral and neurophysiological measures. Correlation coefficients ranged from *r* = 0.492 to *r* = 0.864 (all *p* < .05). Corresponding scatter plots and correlation coefficients for all measures and conditions are shown in Fig. 6.

## 4 Discussion

The present study investigated the mechanisms of selective spatial attention in a two-speaker cocktail-party scenario, contrasting behavioral and electrophysiological results from a real and a virtual environment. Specifically, we examined how variations in the spatial configuration of target and distractor speakers along the azimuth or depth dimension, as well as the presence of visual cues, influence task performance. We analyzed target detection accuracy and reaction times and applied drift-diffusion modeling to characterize the dynamics of evidence accumulation underlying participants’ responses. At the neural level, we focused on the P1-N1-P2 complex as a measure of early auditory processing and on the CNV as a marker of preparatory attention. Ultimately, we assessed whether neural and behavioral findings obtained in a virtual environment matched those from a real-world laboratory setting. To this end, the experiment was conducted both in a traditional reverberant laboratory and in a virtual replication, designed to match the auditory and visual characteristics of the real setup. Establishing such comparability is essential for validating VR as a tool to study auditory attention under more naturalistic and ecologically relevant conditions.

### 4.1 Behavioral results: slower and less accurate decisions in virtual environment and along the depth dimension

Participants performed generally well across all experimental conditions, but performance varied systematically with environment, stimulation, and spatial configuration of the target and distractor stimuli. First, target detection accuracy and reaction times were both affected by the environment: Participants responded more accurately and faster in the real than in the virtual environment. This likely reflects reduced spatial fidelity in the virtual environment, where auditory cues may not fully replicate all details of natural sound perception. Factors such as headphone-based reproduction, the use of static binaural rendering, and limitations in the reproduction of distance cues may have contributed to the observed performance differences between environments (Lombera et al., 2025; Mali and Kavassery Venkateswaran, 2026). As a result, separating target from distractor speakers required greater effort, leading to slower and less efficient target detection. Comparable impairments in performance under VR conditions have been demonstrated across domains such as visual perception, eye-hand coordination, and action control (Harris et al., 2019; Kimura et al., 2017; Lavoie et al., 2024).

Performance improved when visual patterns were added to the auditory cues, particularly in the real environment, highlighting the role of multisensory integration in supporting spatial discrimination. This is consistent with Marucci et al. (2021), who demonstrated that multisensory stimuli reduce cognitive workload, enhance performance, and improve processing speed under high perceptual load in VR. In our study, this advantage was most pronounced when both the target and distractor were presented along the depth dimension, suggesting that visual cues can partially compensate for the limited reliability of auditory distance cues by reducing perceptual uncertainty (Pichora-Fuller, 2008; Teder-Sälejärvi et al., 2005). Specifically, visual depth cues provide robust spatial information that can enhance auditory spatial perception (Anderson and Zahorik, 2011; Calcagno et al., 2012; Zahorik and Wightman, 2001), underscoring the critical role of vision in supporting accurate spatial localization under challenging listening conditions.

The most pronounced effects were observed for the different target-distractor combinations. Participants showed highest accuracy and fastest reaction times when both target and distractor stimuli were presented along the azimuth dimension. This pattern is consistent with our expectations, as lateralized stimuli provide stronger and more salient binaural cues (e.g., ITDs, ILDs), which facilitate their perceptual separation (Blauert, 1996). The effect that participants showed intermediate performance in mixed-dimension trials, where the target and distractor were presented along different spatial dimensions, can be explained by the spatial arrangement of the stimuli in our experimental setup. In these trials, the angular separation between an azimuth and a depth position was smaller than between two stimuli both presented in azimuth, even when their distances to the listener differed. This is consistent with previous research showing that listeners' ability to discriminate between sound sources declines when the sources are spatially close and improves as the distance between them increases (e.g., Best et al., 2004; Sakamoto and Tamakawa, 2023). Notably, in depth-only trials, participants had to rely primarily on distance-related cues, which are inherently less salient and reliable than binaural cues, making spatial discrimination considerably more challenging (Brungart and Simpson, 2002; Kolarik et al., 2016; Lavandier & Prud'homme, 2025; Zahorik, 2002; Zahorik et al., 2005). Consistent with this and as expected, these trials proved the most demanding, showing the lowest accuracy and the longest reaction times.

Drift-diffusion modeling validated the behavioral results and offered further understanding of the underlying decision-making processes. Slower and less accurate responses in the virtual environment and in depth-only trials were largely associated with reduced drift rates, reflecting slower accumulation of sensory evidence rather than changes in response caution (boundary separation) or sensory-motor processing (non-decision time). Mixed-dimension trials showed intermediate drift rates, consistent with their intermediate performance. Interaction effects were generally weak, with only a modest decrease in drift rate for the combination of the virtual environment and audio-visual condition, suggesting that visual cues offer limited additional benefit in the virtual environment.

Taken together, the experimental environment, the modality of the stimuli, and their spatial configuration shape behavioral task performance. The virtual environment, particularly for depth-only trials, seems to enforce additional perceptual and cognitive demands, slowing evidence accumulation and reducing accuracy. Audio-visual cues can partially compensate for these challenges, especially when auditory distance information is limited, while azimuth stimuli consistently enable faster and more accurate responses.

### 4.2 Early sensory ERPs: P2 shows sensitivity to environment and audio-visual cues

Analysis of early sensory ERPs revealed distinct patterns across the P1-N1-P2 complex. P1 amplitudes were largely unaffected by environment, stimulation, or spatial configuration of the target and distractor, and there was no evidence for meaningful interaction effects. Similarly, N1 amplitudes showed little modulation by these factors, indicating that early sensory processing was generally stable across real and virtual environments (as also observed in Stodt et al., 2025), auditory-only and audio-visual conditions, and different spatial configurations of the target and distractor stimuli.

In contrast, P2 amplitudes were modulated by all experimental factors. Notably, amplitudes were larger in the real than in the virtual environment. A similar tendency for an effect of environment was also reported in a previous study using a change-detection task in a comparable experimental setup (Stodt et al., 2025). The P2 is thought to reflect higher-order auditory processing and is typically associated with activity in fronto-central auditory association cortices, including inferior frontal regions, which have been linked to target detection, attentional allocation, and early stimulus evaluation (Picton and Hillyard, 1974; Potts, 2004). These regions are part of a broader auditory attention network involved in the integration and selection of behaviorally relevant sound information (Watkins et al., 2007). From a neurophysiological perspective, this pattern is broadly consistent with engagement of distributed auditory-attentional networks supporting the selection of behaviorally relevant sound information (Alain et al., 2001). Its enhancement in the real environment likely reflects more effective target identification under natural listening conditions, as supported by the behavioral results. By contrast, the virtual environment may impose additional cognitive demands, for instance because spatial cues are less precise or less familiar. Such differences could arise from the use of generic rather than individualized BRIRs, which is discussed further in the limitations section, and may contribute to the comparatively smaller P2 amplitudes observed.

Second, P2 amplitudes were higher in the audio-visual than in the auditory-only condition, reflecting enhanced attentional allocation in the presence of congruent visual cues, in line with the improved behavioral performance observed for audio-visual trials. This effect is consistent with multisensory integration processes indexed by fronto-central electrophysiological activity, reflecting enhanced attentional selection when congruent visual information is available (Liu et al., 2025; Talsma and Woldorff, 2005; Zou et al., 2017).

Finally, P2 amplitudes were largest for trials in which both stimuli were presented along the depth dimension, i.e., the condition with greater spatial separation difficulty, independent of the type of stimulation. Although larger P2 amplitudes have previously been linked to easier tasks (Cranford et al., 2004; Picton and Hillyard, 1974), this effect may reflect differences in attentional demands rather than task difficulty per se. In our setup, the environment and type of stimulation did not vary from trial to trial but were presented blockwise or on different testing days, whereas the spatial arrangement of individual trials varied randomly. In the present paradigm, spatial configurations varied unpredictably across trials, requiring flexible, trial-by-trial allocation of spatial attention. Under these conditions, stimuli that are more difficult to spatially segregate place greater demands on attentional resources, consistent with interpretations of P2 as an index of top-down attentional modulation during early auditory processing (C. Zhang et al., 2019). Neurophysiologically, this is in line with increased recruitment of fronto-parietal attentional control networks, which support spatial selection and prioritization under high perceptual demand (Falkenberg et al., 2011; Westerhausen et al., 2010).

In summary, while early sensory responses (P1, N1) were mostly invariant, P2 was clearly influenced by environment, stimulation, and the spatial distribution of the stimuli. This suggests that P2 reflects the allocation of attentional resources and the involvement of top-down processes in processing more challenging auditory scenes, particularly when sound sources are close together or presented along the depth dimension. Overall, the observed scalp distributions, characterized by a stable fronto-central P2 across conditions, further support the interpretation of the P2 as reflecting interactions between auditory processing systems and higher-order fronto-parietal control networks rather than purely early sensory processing stages.

### 4.3 CNV is sensitive to spatial speaker configuration, not environment or modality

The CNV has a long-standing history in cognitive neuroscience as a marker of anticipatory neural activity, reflecting processes such as expectancy, response preparation, and attentional orienting (Brunia et al., 2011; Brunia and van Boxtel, 2001; Kononowicz and Penney, 2016; Walter et al., 1964). In our study, CNV amplitude was primarily influenced by the spatial configuration of the stimuli. Specifically, the largest (most negative) amplitudes were observed when both target and distractor were presented in azimuth, whereas depth-only trials elicited the smallest (least negative) amplitudes. This pattern is consistent with previous findings showing that larger CNV amplitudes precede easier rather than more difficult discrimination tasks (Perdok and Gaillard, 1979), and that the CNV is generally reduced when selective attention to task-relevant stimuli is impaired by challenging distractors (McCallum and Walter, 1968; Travis and Tecce, 1998). Together, these results indicate that the CNV reflects anticipatory attention in proportion to task demands: Lateralized azimuth stimuli provide stronger and more salient binaural cues that facilitate perceptual separation, while depth-only stimuli depend on subtler distance cues, which increase attentional demands and make discriminating target from distractor more challenging.

Further, CNV amplitude has been shown to correlate with task performance, with larger amplitudes associated with higher accuracy (Wöstmann et al., 2015). In line with this, azimuth trials in our study elicited both the highest behavioral accuracy and the largest CNV amplitudes, whereas depth-only trials showed reduced accuracy and attenuated CNV responses. These observations support the view that the CNV not only indexes preparatory attention but also reflects enhanced excitability in task-relevant cortical networks, thereby facilitating more efficient processing of expected stimuli (Raichle, 2011). From a neurophysiological perspective, the observed CNV is consistent with activity in fronto-central preparatory control systems, including medial frontal and premotor regions, which are known to support anticipatory attention and response preparation (Gómez et al., 2007). The stable fronto-central distribution observed across conditions further supports this interpretation and aligns with the typical scalp topography of the CNV.

Overall, the CNV dynamically tracks the expected difficulty of the task, increasing when spatial cues are salient and discriminable and decreasing when stimuli are harder to separate, indicating a robust neural measure of anticipatory attention and task preparation. Importantly, neither the experimental environment nor the presence of visual cues significantly modulated CNV amplitude. This suggests that anticipatory processes remain stable across sensory and environmental contexts and are primarily driven by the spatial demands of the task rather than by multisensory integration. The high correspondence of CNV effects between real and virtual environments further supports its suitability as a robust measure of selective spatial attention in VR, strengthening the validity of VR-based research.

### 4.4 Cross-environment correspondence of behavioral and neurophysiological measures

Beyond the specific effects of environment, stimulation, and target-distractor combination discussed above, the primary aim of the present study was to assess the correspondence between real and virtual environments. Across all behavioral and neural measures, significant positive correlations were observed between individual outcomes obtained in the real and virtual environments. These correlations ranged from moderate to strong and were evident for both auditory-only and audio-visual stimulation conditions. Thus, participants who showed relatively higher or lower performance levels in the real environment tended to show similar relative values in the virtual environment, and comparable associations were observed for the ERP measures.

These findings should be considered alongside the observed mean differences between environments. Although participants generally responded more accurately and faster in the real environment, and some ERP components differed in amplitude between environments, these effects reflect systematic differences in overall performance and cortical processing rather than changes in the relative ranking of individuals. In contrast, the positive cross-environment relationships indicate that interindividual differences were preserved, suggesting stable patterns of performance and neural processing across settings.

Together, these findings indicate that the virtual environment reproduces key aspects of individual variability observed in the real environment. This pattern suggests that the virtual setup captures key aspects of the underlying mechanisms of selective spatial attention, despite quantitative differences in overall performance. Accordingly, these results support the use of virtual environments for investigating selective spatial attention on an interindividual level under controlled yet ecologically relevant conditions.

### 4.5 Limitations and outlook

Some limitations of the experimental setup must be acknowledged. First, lower accuracy and longer reaction times in the virtual environment likely reflect the increased difficulty in discriminating the spatial positions of the two active speakers. In our study, generalized BRIRs were used to simulate the auditory scenes, meaning that spatial auditory cues were not individualized for each listener, which may have contributed to reduced spatial precision in the virtual environment. Available evidence suggests that non-individualized BRIRs can provide useable spatial cues for sound localization, with no clear advantage of individualized over non-individualized configurations for distance perception (Berger et al., 2018; Blau et al., 2021; Lombera et al., 2025). Several studies on azimuth localization suggest benefits of individualized HRTFs, with some reporting improved localization accuracy (e.g., Andersen et al., 2021; Jenny and Reuter, 2020). However, other studies have found only small or non-significant effects, often with specific improvements in reducing front-back confusions (e.g., Oberem et al., 2018; Wenzel et al., 1993). Previous work further suggests that dynamic spatial updating may have a greater influence on localization performance than HRTF individualization alone (Oberem et al., 2018). A more critical limitation of the present study is therefore the use of static binaural rendering without dynamic spatial updating, which likely reduced spatial fidelity compared to natural listening.

Another limitation of the current study is the static setup of the auditory scenes. In natural multi-talker environments, speakers move their heads, shift their orientation, and vary their speech intensity, while listeners can also move their heads to focus on a specific speaker. In the present study, participants’ heads were stabilized with a chin rest in both environments, and virtual sound sources were presented from fixed positions without dynamic updating. Consequently, the virtual environment did not provide several cues that are available during natural listening and that may facilitate speaker segregation, particularly in depth.

The use of a static setup was chosen to ensure a controlled comparison between real and virtual environments and to maintain stable EEG recordings throughout the experiment, as head movements can increase muscle artifacts and affect electrode stability. Nevertheless, the absence of dynamic binaural cues may have contributed to the behavioral differences observed between environments and limits the ecological validity of the virtual setup. Future studies should therefore investigate whether dynamic binaural rendering and head tracking further improve the correspondence between real and virtual listening environments.

Finally, the current study employed only two discrete depth positions, which limited both the range and resolution of distance cues available to the participants. Expanding the number of depth positions in future studies would provide a more continuous and nuanced representation of auditory distance, allowing for more precise spatial judgments and a better approximation of real-world listening conditions.

## 5 Conclusion

Our findings reveal that behavioral and neural responses in a two-speaker selective attention task are differentially affected by the experimental environment, stimulus modality, and spatial configuration of the speakers. Participants responded more slowly and less accurately in the virtual environment and in depth-only trials, whereas P2 amplitudes were influenced by multiple factors: they were larger in the real than in virtual environment, larger for auditory-only than audio-visual stimulation, and modulated by the spatial configuration of target and distractor speakers, reflecting attentional allocation sensitive to task difficulty. CNV amplitudes, reflecting preparatory neural activity, were primarily modulated by spatial configuration. Importantly, CNV effects were consistent across both real and virtual environments and unaffected by the presence of visual cues, indicating that anticipatory neural processes underlying selective spatial attention are robust across experimental setups. Beyond these condition-based effects, the cross-environment analyses further demonstrated significant positive associations between behavioral performance and neural measures obtained in the real and virtual environments. Importantly, participants who showed higher performance and stronger neural responses in the real environment also exhibited corresponding patterns in the virtual environment, indicating preserved interindividual variability across settings.

These findings suggest that, despite additional perceptual and cognitive demands, VR reliably captures core behavioral and neural mechanisms of selective spatial attention. With careful design - such as improved distance cues and dynamic scenes - VR can serve as an ecologically valid platform for studying these processes. These findings also have practical implications: VR allows the creation of realistic yet controlled auditory scenarios that are difficult to reproduce in real-world settings, such as busy classrooms or working environments. Furthermore, understanding how multisensory cues affect behavioral and neural performance can inform the design of auditory training programs and assistive systems, such as driving or alerting systems, where visual or other sensory information supports accurate sound localization. Overall, these results highlight the potential of VR not only as a research tool but also as a platform for developing and testing interventions in complex, real-world environments.

## Declaration of generative AI and AI-assisted technologies in the manuscript preparation process

During the preparation of this work, the authors used ChatGPT (OpenAI) to assist with rephrasing, smoothing, and shortening text for clarity and fluency. After using this tool, the authors reviewed and edited the content as needed and take full responsibility for the content of the published article.

## CRediT authorship contribution statement

**Benjamin Stodt:** Conceptualization, Data curation, Formal analysis, Investigation, Methodology, Project administration, Software, Validation, Visualization, Writing – original draft, Writing – review & editing. **Daniel Neudek:** Conceptualization, Methodology, Software, Writing – review & editing. **Rainer Martin:** Conceptualization, Funding acquisition, Methodology, Resources, Supervision, Writing – review & editing. **Edmund Wascher:** Resources, Writing – review & editing. **Stephan Getzmann:** Conceptualization, Funding acquisition, Methodology, Supervision, Writing – review & editing.

## Declaration of competing interest

The authors declare the following financial interests/personal relationships which may be considered as potential competing interests:Stephan Getzmann, Rainer Martin reports financial support was provided by German Research Foundation. If there are other authors, they declare that they have no known competing financial interests or personal relationships that could have appeared to influence the work reported in this paper.

## Data Availability

Data will be made available on request.

## References

[bib1] Alain C., Arnott S.R., Picton T.W. (2001). Bottom-up and top-down influences on auditory scene analysis: evidence from event-related brain potentials. J. Exp. Psychol. Hum. Percept. Perform..

[bib2] Alain C., Arnott S.R. (2000). Selectively attending to auditory objects. Front. Biosci..

[bib3] Andersen J.S., Miccini R., Serafin S., Spagnol S. (2021). Evaluation of individualized HRTFs in a 3D shooter game. 2021 Immersive and 3D Audio: From Architecture to Automotive (I3DA).

[bib4] Anderson P.W., Zahorik P. (2011). Auditory and visual distance estimation. Proc. Meet. Acoust..

[bib5] Arnott S.R., Bardouille T., Ross B., Alain C. (2011). Neural generators underlying concurrent sound segregation. Brain Res..

[bib6] Audacity-Team (2024). https://www.audacityteam.org.

[bib7] Aydin M., Carpenelli A.L., Lucia S., Di Russo F. (2022). The dominance of anticipatory prefrontal activity in uncued sensory-motor tasks. Sensors.

[bib8] Balan O., Moldoveanu A., Moldoveanu F., Morar A., Ivascu S. (2017). 2017 40th International Conference on Telecommunications and Signal Processing (TSP).

[bib9] Balboa A., Cuesta A., Alvear D. (2024). Virtual experiments yield comparable results to physical experiments on evacuation decision-making. J. Phys., Conf. Ser..

[bib10] Bebko A.O., Troje N.F. (2020). bmlTUX: design and control of experiments in virtual reality and beyond. I-Perception.

[bib11] Begault D.R., Wenzel E.M., Anderson M.R. (2001). Direct comparison of the impact of head tracking, reverberation, and individualized head-related transfer functions on the spatial perception of a virtual speech source. J. Audio Eng. Soc..

[bib12] Berger C.C., Gonzalez-Franco M., Tajadura-Jiménez A., Florencio D., Zhang Z. (2018). Generic HRTFs may be good enough in virtual reality. Improving source localization through cross-modal plasticity. Front. Neurosci..

[bib13] Best V., Ozmeral E.J., Shinn-Cunningham B.G. (2007). Visually-guided attention enhances target identification in a complex auditory scene. J. Assoc. Res. Otolaryngol..

[bib14] Best V., van Schaik A., Carlile S. (2004). Separation of concurrent broadband sound sources by human listeners. J. Acoust. Soc. Am..

[bib15] Bidelman G.M., Bernard F., Skubic K. (2025). Hearing in categories and speech perception at the “cocktail party.”. PLoS One.

[bib16] Blau M., Budnik A., Fallahi M., Steffens H., Ewert S.D., Van De Par S. (2021). Toward realistic binaural auralizations - perceptual comparison between measurement and simulation-based auralizations and the real room for a classroom scenario. Acta Acustica.

[bib17] Blauert J. (1996).

[bib18] Bregman A.S. (1990).

[bib19] Bregman A.S., Liao C., Levitan R. (1990). Auditory grouping based on fundamental frequency and formant peak frequency. Can. J. Psychol..

[bib20] Breuer Carolin, Loh Karin, Leist Larissa, Fremerey Stephan, Raake Alexander, Klatte Maria, Fels Janina (2022). Examining the Auditory Selective Attention Switch in a Child-Suited Virtual Reality Classroom Environment. International Journal of Environmental Research and Public Health.

[bib21] Bronkhorst A.W. (2015). The cocktail-party problem revisited: early processing and selection of multi-talker speech. Atten. Percept. Psychophys..

[bib22] Brungart D.S., Simpson B.D. (2002). The effects of spatial separation in distance on the informational and energetic masking of a nearby speech signal. J. Acoust. Soc. Am..

[bib23] Brunia C.H.M., van Boxtel G.J.M., Böcker K.B.E., Kappenman E.S., Luck S.J. (2011). The Oxford Handbook of Event-Related Potential Components.

[bib24] Brunia C.H.M., van Boxtel G.J.M. (2001). Wait and see. Int. J. Psychophysiol..

[bib25] Calcagno E.R., Abregú E.L., Eguía M.C., Vergara R. (2012). The role of vision in auditory distance perception. Perception.

[bib26] Carlyon R.P. (2004). How the brain separates sounds. Trends Cognit. Sci..

[bib27] Cherry E.C. (1953). Some experiments on the recognition of speech, with one and with two ears. J. Acoust. Soc. Am..

[bib28] Cranford J.L., Rothermel A.K., Walker L., Stuart A., Elangovan S. (2004). Effects of discrimination task difficulty on N1 and P2 components of late auditory evoked potential. J. Am. Acad. Audiol..

[bib29] Darwin C.J. (2008). Listening to speech in the presence of other sounds. Philos. Trans. R. Soc. Lond. B Biol. Sci..

[bib30] de la Rosa S., Breidt M. (2018). Virtual reality: a new track in psychological research. Br. J. Psychol..

[bib31] Delorme A., Makeig S. (2004). EEGLAB: an open source toolbox for analysis of single-trial EEG dynamics including independent component analysis. J. Neurosci. Methods.

[bib32] Eggermont J.J., Ponton C.W. (2002). The neurophysiology of auditory perception: from single units to evoked potentials. Audiol. Neurootol..

[bib33] Falkenberg L.E., Specht K., Westerhausen R. (2011). Attention and cognitive control networks assessed in a dichotic listening fMRI study. Brain Cognit..

[bib34] Feng Y., Liu K., Zhang R., Wang H., Gao Y., Xu Z., Yang J. (2025). Effect of visually cued spatial and temporal attention on audiovisual stimuli processing: an event-related potentials study. Front. Psychol..

[bib35] Friedman D., Simpson G., Hamberger M. (1993). Age‐related changes in scalp topography to novel and target stimuli. Psychophysiology.

[bib36] Getzmann S., Golob E.J., Wascher E. (2016). Focused and divided attention in a simulated cocktail-party situation: ERP evidence from younger and older adults. Neurobiol. Aging.

[bib37] Getzmann S., Lewald J., Falkenstein M. (2014). Using auditory pre-information to solve the cocktail-party problem: electrophysiological evidence for age-specific differences. Front. Neurosci..

[bib38] Getzmann S., Falkenstein M. (2011). Understanding of spoken language under challenging listening conditions in younger and older listeners: a combined behavioral and electrophysiological study. Brain Res..

[bib39] Getzmann S., Näätänen R. (2015). The mismatch negativity as a measure of auditory stream segregation in a simulated “cocktail-party” scenario: effect of age. Neurobiol. Aging.

[bib40] Getzmann S., Wascher E. (2017). Visually guided auditory attention in a dynamic “cocktail-party” speech perception task: ERP evidence for age-related differences. Hear. Res..

[bib41] Gómez C.M., Flores A., Ledesma A. (2007). Fronto-parietal networks activation during the contingent negative variation period. Brain Res. Bull..

[bib42] Grunwald T., Boutros N.N., Pezer N., Von Oertzen J., Fernández G., Schaller C., Elger C.E. (2003). Neuronal substrates of sensory gating within the human brain. Biol. Psychiatry.

[bib43] Guastamacchia A., Rosso R.G., Puglisi G.E., Riente F., Shtrepi L., Pellerey F., Astolfi A. (2025). Subjective and objective validation of a virtual reality system as a tool for studying speech intelligibility in architectural spaces. J. Acoust. Soc. Am..

[bib44] Harris D.J., Buckingham G., Wilson M.R., Vine S.J. (2019). Virtually the same? How impaired sensory information in virtual reality may disrupt vision for action. Exp. Brain Res..

[bib45] Hawley M.L., Litovsky R.Y., Colburn H.S. (1999). Speech intelligibility and localization in a multi-source environment. J. Acoust. Soc. Am..

[bib46] Haykin S., Chen Z. (2005). The cocktail party problem. Neural Comput..

[bib47] Hillyard S.A., Hink R.F., Schwent V.L., Picton T.W. (1973). Electrical signs of selective attention in the human brain. Science.

[bib48] Hohmann V., Paluch R., Krueger M., Meis M., Grimm G. (2020). The virtual reality lab: realization and application of virtual sound environments. Ear Hear..

[bib49] Ihlefeld A., Shinn-Cunningham B. (2008). Spatial release from energetic and informational masking in a divided speech identification task. J. Acoust. Soc. Am..

[bib50] JASP Team (2025). *JASP* (Version 0.95). https://jasp-stats.org/.

[bib51] Jeffreys H. (1961).

[bib52] Jenny C., Reuter C. (2020). Usability of individualized head-related transfer functions in virtual reality: empirical study with perceptual attributes in sagittal plane sound localization. JMIR Serious Games.

[bib53] Kalantari S., Mostafavi A., Xu T.B., Lee A.S., Yang Q. (2024). Comparing spatial navigation in a virtual environment vs. an identical real environment across the adult lifespan. Comput. Hum. Behav..

[bib54] Kalantari S., Rounds J.D., Kan J., Tripathi V., Cruz-Garza J.G. (2021). Comparing physiological responses during cognitive tests in virtual environments vs. in identical real-world environments. Sci. Rep..

[bib55] Kass R.E., Raftery A.E. (1995). Bayes factors. J. Am. Stat. Assoc..

[bib56] Kaya E.M., Elhilali M. (2017). Modelling auditory attention. Philos. Trans. R. Soc. Lond. B Biol. Sci..

[bib57] Keidser G., Naylor G., Brungart D.S., Caduff A., Campos J., Carlile S., Carpenter M.G., Grimm G., Hohmann V., Holube I., Launer S., Lunner T., Mehra R., Rapport F., Slaney M., Smeds K. (2020). The quest for ecological validity in hearing science: what it is, why it matters, and how to advance it. Ear Hear..

[bib58] Kidd G., Mason C.R., Gallun F.J. (2005). Combining energetic and informational masking for speech identification. J. Acoust. Soc. Am..

[bib59] Kimura K., Reichert J.F., Olson A., Pouya O.R., Wang X., Moussavi Z., Kelly D.M. (2017). Orientation in virtual reality does not fully measure up to the real-world. Sci. Rep..

[bib60] Kolarik A.J., Moore B.C.J., Zahorik P., Cirstea S., Pardhan S. (2016). Auditory distance perception in humans: a review of cues, development, neuronal bases, and effects of sensory loss. Atten. Percept. Psychophys..

[bib61] Kononowicz T.W., Penney T.B. (2016). The contingent negative variation (CNV): timing isn't everything. Curr. Opin. Behav. Sci..

[bib62] Lange K. (2013). The ups and downs of temporal orienting: a review of auditory temporal orienting studies and a model associating the heterogeneous findings on the auditory N1 with opposite effects of attention and prediction. Front. Hum. Neurosci..

[bib63] Lavandier M., Prud’homme L. (2025). Reverberation cues underlying virtual auditory distance perception for a frontal source. J. Acoust. Soc. Am..

[bib64] Lavoie E., Hebert J.S., Chapman C.S. (2024). Comparing eye–hand coordination between controllermediated virtual reality, and a real-world object interaction task. J. Vis..

[bib65] Lewald J., Hanenberg C., Getzmann S. (2016). Brain correlates of the orientation of auditory spatial attention onto speaker location in a “cocktail-party” situation. Psychophysiology.

[bib66] Lewald J., Getzmann S. (2015). Electrophysiological correlates of cocktail-party listening. Behav. Brain Res..

[bib67] Liesefeld H.R. (2018). Estimating the timing of cognitive operations with MEG/EEG latency measures: a primer, a brief tutorial, and an implementation of various methods. Front. Neurosci..

[bib68] Lin W.-Y., Venkatakrishnan R., Venkatakrishnan R., Babu S.V., Pagano C., Lin W.-C. (2024). 2024 IEEE Conference Virtual Reality and 3D User Interfaces (VR).

[bib69] Liu W., Sun J., Yang J., Tang X., Lu S. (2025). 2025 19th International Conference on Complex Medical Engineering (CME).

[bib70] Lombera E.N., Cerviño J., Piceda L.B., Viskovic M., Vergara R.O. (2025). Comparison of perceived auditory distance between real and virtual sound sources. Appl. Acoust..

[bib71] Lopez-Calderon J., Luck S.J. (2014). ERPLAB: an open-source toolbox for the analysis of event-related potentials. Front. Hum. Neurosci..

[bib72] Loushy Kay T., Been E., Pick C.G. (2025). A novel reaction time assessment in virtual reality: advantages over computerized tests. Behav. Res. Methods.

[bib73] Majdak P., Goupell M.J., Laback B. (2010). 3-D localization of virtual sound sources: effects of visual environment, pointing method, and training. Atten. Percept. Psychophys..

[bib74] Mali H., Kavassery Venkateswaran N. (2026). Auditory spatial assessment in real and virtual environments: a scoping review of current evidence and future directions. Egypt. J. Otolaryngol..

[bib75] Marucci M., Di Flumeri G., Borghini G., Sciaraffa N., Scandola M., Pavone E.F., Babiloni F., Betti V., Aricò P. (2021). The impact of multisensory integration and perceptual load in virtual reality settings on performance, workload and presence. Sci. Rep..

[bib76] Masiero B., Fels J. (2011). Perceptually robust headphone equalization for binaural reproduction. Proc. AES Conv.

[bib77] Mathiak K., Hertrich I., Kincses W.E., Riecker A., Lutzenberger W., Ackermann H. (2003). The right supratemporal plane hears the distance of objects: neuromagnetic correlates of virtual reality. Neuroreport.

[bib78] McCallum W.C., Walter W.G. (1968). The effects of attention and distraction on the contingent negative variation in normal and neurotic subjects. Electroencephalogr. Clin. Neurophysiol..

[bib79] Minderer M., Harvey C.D., Donato F., Moser E.I. (2016). Virtual reality explored. Nature.

[bib80] Møller H. (1992). Fundamentals of binaural technology. Appl. Acoust..

[bib81] Moraes A.N., Flynn R., Hines A., Murray N. (2023). 2023 15th International Conference on Quality of Multimedia Experience (Qomex).

[bib82] Noble W., Perrett S. (2002). Hearing speech against spatially separate competing speech versus competing noise. Percept. Psychophys..

[bib83] Näätänen R., Picton T. (1987). The N1 wave of the human electric and magnetic response to sound: a review and an analysis of the component structure. Psychophysiology.

[bib84] Oberem J., Richter J.-G., Setzer D., Seibold J., Koch I., Fels J. (2018). Experiments on localization accuracy with non-individual and individual HRTFs comparing static and dynamic reproduction methods. Proceedings of the DAGA.

[bib85] Pappalettera C., Miraglia F., Cacciotti A., Nucci L., Tufo G., Rossini P.M., Vecchio F. (2024). The impact of virtual reality and distractors on attentional processes: insights from EEG. Pflugers Arch. Eur. J. Physiol..

[bib86] Paquier M., Côté N., Devillers F., Koehl V. (2016). Interaction between auditory and visual perceptions on distance estimations in a virtual environment. Appl. Acoust..

[bib87] Parseihian G., Jouffrais C., Katz B.F.G. (2014). Reaching nearby sources: comparison between real and virtual sound and visual targets. Front. Neurosci..

[bib88] Parsons T.D. (2015). Virtual reality for enhanced ecological validity and experimental control in the clinical, affective and social neurosciences. Front. Hum. Neurosci..

[bib89] Perdok J., Gaillard A.W.K. (1979). The terminal CNV and stimulus discriminability in motor and sensory tasks. Biol. Psychol..

[bib90] Piceda L.B., Lombera E., Vergara R. (2025). Localization of auditory sources spatialized in virtual environments. Musica Hodie.

[bib91] Pichora-Fuller M.K. (2008). Use of supportive context by younger and older adult listeners: balancing bottom-up and top-down information processing. Int. J. Audiol..

[bib92] Picton T.W., Hillyard S.A. (1974). Human auditory evoked potentials. II: effects of attention. Electroencephalogr. Clin. Neurophysiol..

[bib93] Pion-Tonachini L., Kreutz-Delgado K., Makeig S. (2019). ICLabel: an automated electroencephalographic independent component classifier, dataset, and website. Neuroimage.

[bib94] Posit Team (2025). *RStudio: Integrated development environment for R* (Version 2025.05.1). http://www.posit.co.

[bib95] Potts G.F. (2004). An ERP index of task relevance evaluation of visual stimuli. Brain Cognit..

[bib96] Raichle M.E. (2011). The restless brain. Brain Connect..

[bib97] Ramírez M., Müller A., Arend J.M., Himmelein H., Rader T., Pörschmann C. (2024). Speech-in-noise testing in virtual reality. Front. Virtual Real.

[bib98] Ratcliff R. (1978). A theory of memory retrieval. Psychol. Rev..

[bib99] Ratcliff R., Smith P.L., Brown S.D., McKoon G. (2016). Diffusion decision model: current issues and history. Trends Cognit. Sci..

[bib100] Roßkopf S., Muhlberger A., Starz F., Van De Par S., Blau M., Kroczek L.O.H. (2025). Impact of visual virtual scene and localization task on auditory distance perception in virtual reality. IEEE Trans. Vis. Comput. Graph..

[bib101] Rumiński D. (2015). An experimental study of spatial sound usefulness in searching and navigating through AR environments. Virtual Real..

[bib102] Sakamoto S., Tamakawa M. (2023). The effect of angular disparity between sound source sequences on the perceptual organization of auditory objects. J. Acoust. Soc. Am..

[bib103] Shafiro V., Gygi B. (2007). Perceiving the speech of multiple concurrent talkers in a combined divided and selective attention task. J. Acoust. Soc. Am..

[bib104] Shelton B.R., Searle C.L. (1980). The influence of vision on the absolute identification of sound-source position. Percept. Psychophys..

[bib105] Stodt B., Neudek D., Getzmann S., Wascher E., Martin R. (2024). Comparing auditory distance perception in real and virtual environments and the role of the loudness cue: a study based on event-related potentials. Hear. Res..

[bib106] Stodt B., Neudek D., Martin R., Wascher E., Getzmann S. (2025). Age‐related differences in neural correlates of auditory spatial change detection in real and virtual environments. Eur. J. Neurosci..

[bib107] Sussman E.S. (2005). Integration and segregation in auditory scene analysis. J. Acoust. Soc. Am..

[bib108] Sussman E.S. (2017). Auditory scene analysis: an attention perspective. J. Speech Lang. Hear. Res..

[bib109] Talsma D., Woldorff M.G. (2005). Selective attention and multisensory integration: multiple phases of effects on the evoked brain activity. J. Cognit. Neurosci..

[bib110] Tecce J.J., Scheff N.M. (1969). Attention reduction and suppressed direct-current potentials in the human brain. Science.

[bib111] Teder-Sälejärvi W.A., Russo F. Di, McDonald J.J., Hillyard S.A. (2005). Effects of spatial congruity on audio-visual multimodal integration. J. Cognit. Neurosci..

[bib112] The MathWorks Inc (2024). *MATLAB* (Version R2024a). https://www.mathworks.com.

[bib113] Thurley K. (2022). Naturalistic neuroscience and virtual reality. Front. Syst. Neurosci..

[bib114] Travis F., Tecce J.J. (1998). Effects of distracting stimuli on CNV amplitude and reaction time. Int. J. Psychophysiol..

[bib115] Valzolgher Chiara, Capra Sara, Gessa Elena, Rosi Tommaso, Giovanelli Elena, Pavani Francesco (2024). Sound localization in noisy contexts: performance, metacognitive evaluations and head movements. Cognitive Research: Principles and Implications.

[bib116] Voicemaker (2026). Voicemaker*.in*. https://www.voicemaker.in.

[bib117] Walter W.G., Cooper R., Aldridge V.J., McCallum W.C., Winter A.L. (1964). Contingent negative variation: an electric sign of sensori-motor association and expectancy in the human brain. Nature.

[bib118] Watkins S., Dalton P., Lavie N., Rees G. (2007). Brain mechanisms mediating auditory attentional capture in humans. Cerebr. Cortex.

[bib119] Wenzel E.M., Arruda M., Kistler D.J., Wightman F.L. (1993). Localization using nonindividualized head‐related transfer functions. J. Acoust. Soc. Am..

[bib120] Westerhausen R., Moosmann M., Alho K., Belsby S.-O., Hämäläinen H., Medvedev S., Specht K., Hugdahl K. (2010). Identification of attention and cognitive control networks in a parametric auditory fMRI study. Neuropsychologia.

[bib121] Wöstmann M., Schröger E., Obleser J. (2015). Acoustic detail guides attention allocation in a selective listening task. J. Cognit. Neurosci..

[bib122] Zahorik P. (2002). Assessing auditory distance perception using virtual acoustics. J. Acoust. Soc. Am..

[bib123] Zahorik P., Brungart D.S., Bronkhorst A.W. (2005). Auditory distance perception in humans: a summary of past and present research. Acta Acust. United Ac.

[bib124] Zahorik P., Wightman F.L. (2001). Loudness constancy with varying sound source distance. Nat. Neurosci..

[bib125] Zhang C., Tao R., Zhao H. (2019). Auditory spatial attention modulates the unmasking effect of perceptual separation in a “cocktail party” environment. Neuropsychologia.

[bib126] Zhang W., Samarasinghe P., Chen H., Abhayapala T. (2017). Surround by sound: a review of spatial audio recording and reproduction. Appl. Sci..

[bib127] Zou Z., Chau B.K.H., Ting K.-H., Chan C.C.H. (2017). Aging effect on audiovisual integrative processing in spatial discrimination task. Front. Aging Neurosci..

